# A three-step MTOC fragmentation mechanism facilitates bipolar spindle assembly in mouse oocytes

**DOI:** 10.1038/ncomms8217

**Published:** 2015-07-06

**Authors:** Dean Clift, Melina Schuh

**Affiliations:** 1 Medical Research Council, Laboratory of Molecular Biology, Cambridge CB2 0QH, UK

## Abstract

Assembly of a bipolar microtubule spindle is essential for accurate chromosome segregation. In somatic cells, spindle bipolarity is determined by the presence of exactly two centrosomes. Remarkably, mammalian oocytes do not contain canonical centrosomes. This study reveals that mouse oocytes assemble a bipolar spindle by fragmenting multiple acentriolar microtubule-organizing centres (MTOCs) into a high number of small MTOCs to be able to then regroup and merge them into two equal spindle poles. We show that MTOCs are fragmented in a three-step process. First, PLK1 triggers a decondensation of the MTOC structure. Second, BicD2-anchored dynein stretches the MTOCs into fragmented ribbons along the nuclear envelope. Third, KIF11 further fragments the MTOCs following nuclear envelope breakdown so that they can be evenly distributed towards the two spindle poles. Failure to fragment MTOCs leads to defects in spindle assembly, which delay chromosome individualization and congression, putting the oocyte at risk of aneuploidy.

## Abstract (web summary)

Mitotic spindles assemble from two centrosomes, but oocytes lack centrosomes so how their spindles assemble is unclear. Here Clift and Schuh show that multiple acentriolar microtubule-organizing centres fragment in a three-step process to facilitate bipolar spindle assembly in mouse oocytes.

## Graphical abstract

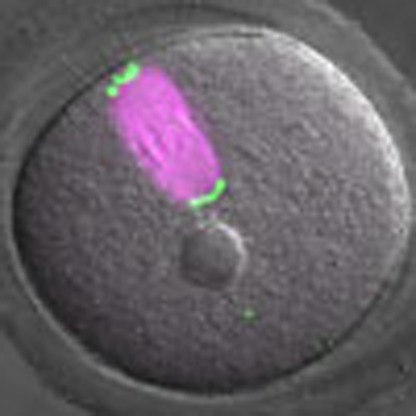

Every time a cell divides, it needs to assemble a bipolar microtubule spindle to accurately segregate its chromosomes. In mammalian somatic cells, spindle bipolarity is determined by the presence of exactly two centrosomes. At the beginning of mitosis, the centrosomes are paired. To ensure that microtubules are nucleated from two distinct poles, the centrosomes start to migrate to opposite sides of the nucleus before nuclear envelope breakdown (NEBD)^1^. After NEBD, the centrosomes assemble a bipolar spindle, which captures the chromosomes and segregates them to opposite spindle poles. Extra centrosomes lead to the formation of multipolar spindles and chromosome segregation errors, and have been suggested to be a key cause of aneuploidy in cancer cells^2^,^3^,^4^. Thus, the number of centrosomes needs to be strictly regulated to ensure that a bipolar spindle assembles. This is achieved by the centrosome cycle, which ensures that centrosomes duplicate only once every cell cycle during the S-phase^5^.

In contrast to somatic cells, mammalian oocytes lack canonical centriole-containing centrosomes^6^. Thus, bipolar spindle assembly needs to be achieved by a different mechanism. In mouse oocytes, spindle microtubules are nucleated by multiple acentriolar microtubule-organizing centres (MTOCs)^7^. These MTOCs contain many of the pericentriolar material components^8^,^9^,^10^ of centrosomes; however, in contrast to centrosomes, they lack centriole pairs at their core^11^. How meiotic spindle bipolarity is achieved with multiple MTOCs remains unknown.

In this study we used quantitative live cell microscopy to investigate how a bipolar spindle is assembled from multiple MTOCs in oocytes. Our data uncover a novel mechanism that facilitates spindle assembly in the absence of centrosomes: the MTOCs exhibit remarkable plasticity and undergo a three-step decondensation and fragmentation process, which facilitates the equal distribution of MTOC material between the two spindle poles. Failure to fragment MTOCs leads to the formation of transient monopolar and asymmetric spindles, resulting in delayed bipolar spindle formation and chromosome congression to the spindle equator. MTOC fragmentation is therefore essential for accurate spindle assembly in the absence of centrosomes in mouse oocytes.

## Results

### MTOCs are fragmented during spindle assembly

**Figure 1:**
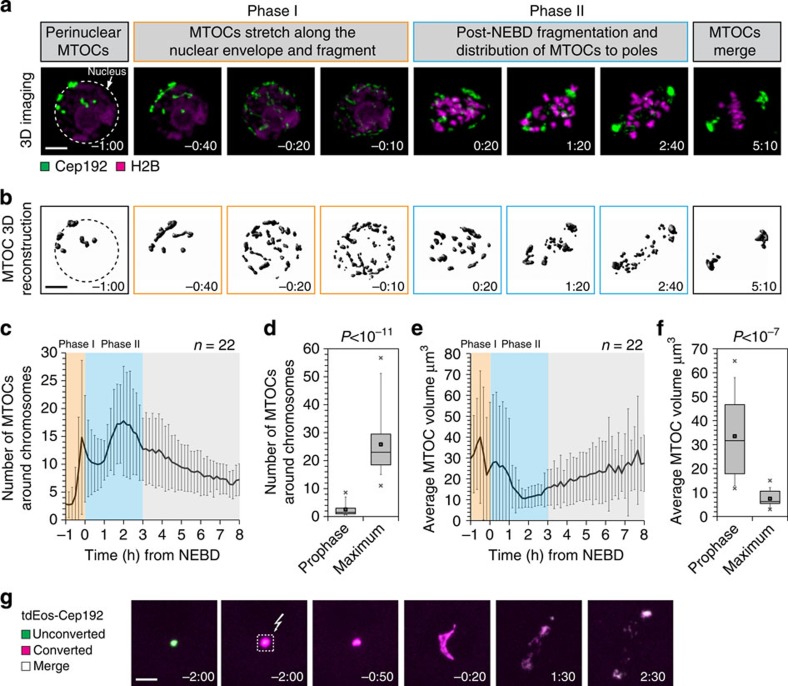
MTOCs fragment in two phases during meiotic spindle assembly. (**a**) 3D time-lapse imaging of MTOCs in live mouse oocytes expressing EGFP-Cep192 (green, MTOCs) and H2B-mRFP1 (magenta, chromosomes). *Z*-projection, 25 sections, every 1.5 μm. Representative example from two independent experiments (22 oocytes in total). (**b**) 3D reconstruction of MTOCs from oocyte shown in **a**. Scale bar, 10 μm. Time, h:min from NEBD. Dashed line outlines the nucleus. (**c**) The mean number of MTOCs surrounding chromosomes plotted over time. Error bars show s.d. (**d**) Box plots of number of MTOCs in prophase and maximum number of MTOCs during spindle assembly. (**e**) The mean volume of MTOCs surrounding chromosomes plotted over time. Error bars show s.d. (**f**) Box plots of volume of MTOCs in prophase and minimum volume of MTOCs during spindle assembly. Data in graphs (**c**–**f**) were obtained from 22 oocytes from two independent experiments. *P* values were calculated with Student’s *t*-test. Orange and blue boxes/shading highlight phases I and II of MTOC fragmentation, respectively. (**g**) 3D time-lapse imaging of MTOCs in live mouse oocytes expressing tdEos-Cep192. MTOCs were photoconverted by irradiation with a 405-nm laser line for 15–20 s in a boxed region containing the MTOCs. Dashed line and jagged arrow indicate the region of photoconversion. Merge of unconverted and converted Cep192 appears white (merge). Scale bar, 10 μm. Time, h:min from NEBD. Representative example from three independent experiments (26 oocytes in total).

To address how spindle bipolarity is achieved from multiple MTOCs, we recorded high-resolution three-dimensional (3D) data sets of MTOCs during spindle assembly in live mouse oocytes. MTOCs were visualized with a tagged version of the pericentriolar material component Cep192 (ref. 12), which we found to be a *bona fide* marker for MTOCs as judged by colocalization with known MTOC components γ-tubulin and Pericentrin (Supplementary Fig. 1). Oocytes assembled a bipolar spindle by first fragmenting the MTOCs into many smaller MTOCs before refocussing them to form the two poles of the meiotic spindle (Fig. 1a; Supplementary Movie 1). To quantify MTOC fragmentation, we reconstructed the MTOCs in proximity of the chromosomes in 3D and measured their number and volume over time (Fig. 1b–f). This revealed that MTOCs were fragmented in two distinct phases: a first phase before NEBD and a second phase after NEBD (Fig. 1c,e; Supplementary Movie 2). During these two phases, the number of MTOCs in proximity of the chromosomes increased from 3±2 to an average maximum of 26±11 (Fig. 1d). Consistent with fragmentation, the average MTOC volume dropped from 30±16 to 7±3 μm^3^ within the same time frame (Fig. 1f). Our quantitative live analysis of MTOCs is in line with an early ultrastructural study that reported an increase in the number of MTOCs between prophase arrest and the onset of meiotic maturation^13^, confirming that our live cell MTOC marker is representative of endogenous MTOC behaviour. As the two spindle poles assembled, the MTOCs merged again, leading to a decrease in MTOC number and an increase in MTOC volume (Fig. 1).

Our data are consistent with MTOC fragmentation (Fig. 1a–f). However, the MTOC fragments we observe could also be generated as a result of the disassembly of large MTOCs and *de novo* formation of many smaller MTOCs. To distinguish between fragmentation and *de novo* formation, we performed a fluorescence pulse-chase experiment to monitor the fate of individual MTOCs during spindle assembly. Cep192 was tagged with the photoconvertible fluorescent protein tdEos, which undergoes irreversible conversion from green to red emission on irradiation with a 405-nm laser line^14^. Individual perinuclear MTOCs were photoconverted before spindle assembly, and both unconverted (green) and converted (magenta) MTOCs were imaged as the spindle assembled (Fig. 1g). We saw gradual recovery of unconverted Cep192 at MTOCs during spindle assembly. This is presumably due to Cep192 turnover and the merging with MTOCs recruited from the cytoplasm^7^, which contain unconverted Cep192. Nonetheless, the large majority of MTOCs detected during spindle assembly contained photoconverted Cep192 (Fig. 1g), suggesting that they arose from the fragmentation of MTOCs present before spindle assembly.

### BicD2-anchored dynein stretches MTOCs on the nuclear surface

**Figure 2:**
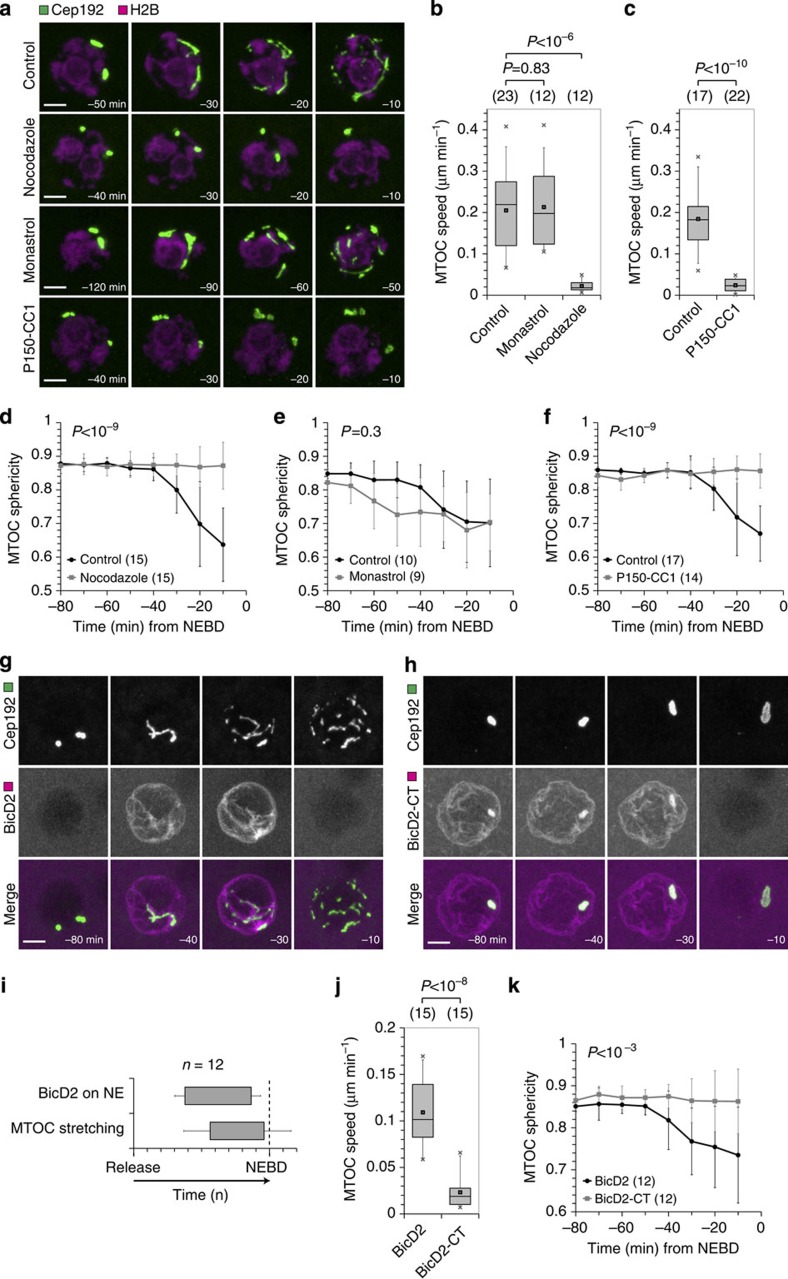
BicD2-anchored dynein stretches MTOCs along the nuclear envelope. (**a**) 3D time-lapse imaging of MTOCs in live mouse oocytes of the indicated condition expressing EGFP-Cep192 (green, MTOCs) and H2B-mRFP1 (magenta, chromosomes). *Z*-projection, 25 sections, every 1.5 μm. Scale bar, 10 μm. Time, minutes from NEBD. Representative examples from two to four independent experiments (>11 oocytes total for each condition). (**b**,**c**) Box plots of speed of MTOC stretching quantified from data sets shown in **a**. Number of oocytes analysed is specified in brackets above each box (aggregation over two to four independent experiments). *P* values were calculated with Student’s *t*-test of the mean values. (**d**–**f**) The mean values for MTOC sphericity quantified from data sets shown in **a** plotted over time. Error bars show s.d. Number of oocytes analysed for each condition is specified in brackets (aggregation over two to four independent experiments). *P* values were calculated with Student’s *t*-test of the mean values for MTOC sphericity at time point before NEBD. (**g**,**h**) 3D Time-lapse imaging of oocytes expressing EGFP-Cep192 (green, MTOCs) and (**g**) mCherry-BicD2 (magenta) or (**h**) mCherry-BicD2-CT (magenta). Z-projection, 25 sections, every 1.5 μm. Scale bar, 10 μm. Time, minutes from NEBD. Representative examples from two independent experiments (12 oocytes in total for each condition). (**i**) Oocytes from **g** were analysed for timing of BicD2 association with nuclear envelope (NE) and MTOC stretching. Time between release from prophase arrest and NEBD was normalized to account for differences in NEBD timing between oocytes. Twelve oocytes from two independent experiments were analysed. Error bars show s.d. (**j**) Box plots of speed of MTOC stretching quantified from data sets shown in **g**,**h**. Number of oocytes analysed is specified in brackets above each box (aggregation over two independent experiments). *P* values were calculated with Student’s *t*-test. (**k**) The mean values for MTOC sphericity quantified from data sets shown in **g**,**h** plotted over time. Error bars show s.d. Number of oocytes analysed for each condition is specified in brackets (aggregation over two independent experiments). *P* value was calculated with Student’s *t*-test of the mean values for MTOC sphericity at time point before NEBD.

We next addressed the mechanism by which MTOCs were fragmented. In phase I, MTOCs were stretched into ribbons along the nuclear envelope (Fig. 1a; Supplementary Movies 1 and 2). In the majority of oocytes (17/22), MTOC stretching proceeded to such an extent that MTOCs fragmented before NEBD. The stretched MTOCs colocalized with microtubules extending along the nuclear envelope (Supplementary Fig. 2). To test whether MTOC stretching is microtubule-dependent, we depolymerized microtubules with nocodazole. Indeed, we found that the MTOCs were no longer stretched along the nuclear envelope (Fig. 2a). This was quantitatively confirmed by measuring the speed of MTOC elongation in the presence and absence of nocodazole (Fig. 2b), and by quantifying the sphericity of the MTOCs: while the stretching reduced MTOC sphericity in control oocytes, MTOC sphericity stayed constant in nocodazole-treated oocytes (Fig. 2d). These results demonstrate that MTOC stretching depends on microtubules on the nuclear envelope, consistent with qualitative observations made in a previous study^15^.

Next, we investigated the molecular mechanism that mediates MTOC stretching. It seemed likely that motor proteins associated with microtubules on the nuclear envelope would drive MTOC stretching. One potential candidate was the plus-end-directed kinesin KIF11 (also known as Kinesin-5 or Eg5). KIF11 crosslinks and slides apart antiparallel microtubules and is essential to separate centrosomes from each other before NEBD^16^,^17^. To test whether KIF11 drives MTOC stretching, we inhibited KIF11 with monastrol^18^. However, neither the speed of MTOC elongation nor the reduction of MTOC sphericity were perturbed (Fig. 2a,b,e). MTOC stretching did occur earlier with respect to NEBD in monastrol-treated oocytes (Fig. 2a,e), although this is likely due to a delay in NEBD onset caused by monastrol treatment (Supplementary Fig. 3). Thus, KIF11 is dispensable for MTOC stretching along the nuclear envelope (Fig. 2a,b,e).

Another important motor protein on the nuclear envelope is dynein. Nuclear envelope-associated dynein is, for instance, involved in nuclear oscillations in neural progenitor cells^19^, positioning of nuclei in myotubes^20^ and has also been implicated in centrosome separation^1^,^21^. To test whether MTOC stretching is dynein-dependent, we inhibited dynein activity in oocytes by expressing P150-CC1, a dominant-negative fragment of dynactin^22^. Strikingly, dynein inhibition completely blocked the stretching of MTOCs along the nuclear envelope (Fig. 2a,c,f). This is in contrast to a previous study that reported MTOC stretching to be independent of dynein^15^. This discrepancy may be explained by insufficient inhibition of dynein by the inhibitor Ciliobrevin-D^23^ that was used in the previous study (Supplementary Fig. 4). Since dynein takes over many functions in a cell^24^, whose inhibition might affect MTOC stretching in an indirect way, we aimed to block dynein function specifically at the nuclear envelope. Dynein is recruited to the nuclear envelope by the adaptor protein BicD2 (refs 21, 25). We found that BicD2 transiently associated with the nuclear envelope while MTOCs were stretched (Fig. 2g,i; Supplementary Movie 3). Consistent with a role for BicD2-recruited dynein in MTOC stretching, BicD2 was enriched at invaginations on the nuclear envelope that contained stretched MTOCs (Fig. 2g). To test the role of nuclear envelope-associated dynein in MTOC stretching directly, we expressed a dominant-negative form of BicD2 (BicD2-CT) that can no longer bind dynein/dynactin^25^. Similar to global dynein inhibition, BicD2-CT expression completely blocked MTOC stretching on the nuclear envelope (Fig. 2h,j,k; Supplementary Movie 3). BicD2-CT specifically inhibited dynein function at the nuclear envelope because the dynein-dependent movement of cytoplasmic MTOCs was unaffected (Supplementary Fig. 5). These data show that a nuclear envelope-associated pool of dynein that is anchored by BicD2 drives MTOC stretching.

### KIF11 drives MTOC fragmentation after NEBD

**Figure 3:**
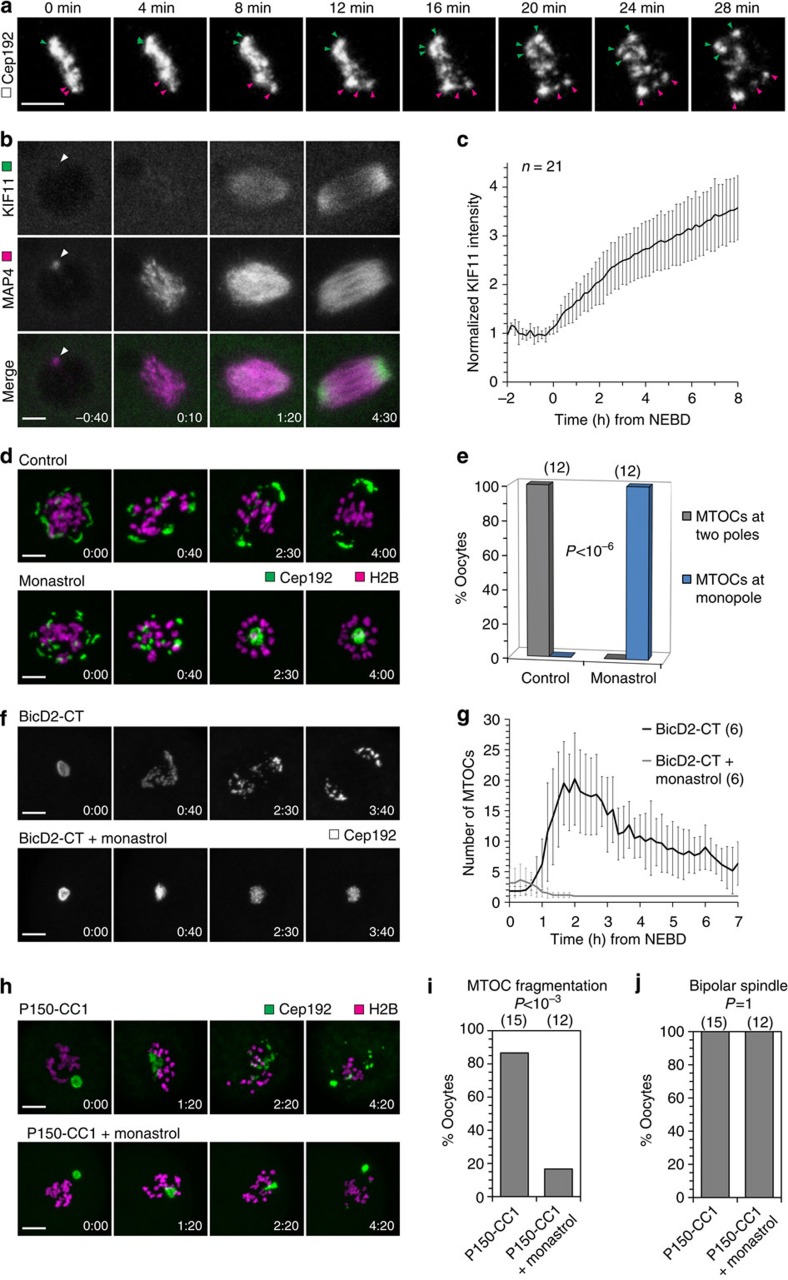
KIF11 fragments MTOCs after NEBD. (**a**) 3D time-lapse imaging of MTOCs after NEBD in oocytes expressing EGFP-Cep192 (green, MTOCs). Twenty-five sections, every 1.5 μm, every 4 min. Scale bar, 5 μm. Representative examples from five independent experiments (27 oocytes in total). Green and magenta arrowheads highlight different MTOC fragmentation events, respectively. (**b**) 3D time-lapse imaging of oocytes expressing KIF11-mEGFP (green, KIF11) and mCherry-MAP4 (magenta, microtubules). Z-projection, seven sections, every 4 μm. Scale bar, 10 μm. Time, h:min from NEBD. Representative example from two independent experiments (21 oocytes in total). (**c**) The mean KIF11-mEGFP intensity within microtubule (mCherry-MAP4) region, normalized to initial value, is plotted over time. Error bars show s.d. (**d**) 3D time-lapse imaging of MTOCs in oocytes expressing EGFP-Cep192 (green, MTOCs) and H2B-mRFP1 (magenta, chromosomes) treated with DMSO (control) or monastrol. Z-projection, 25 sections, every 1.5 μm. Scale bar, 10 μm. Time, h:min from NEBD. Representative examples from two independent experiments. (**e**) MTOC distribution was scored at 6 h after NEBD. Number of oocytes analysed for each condition is specified in brackets (aggregation over two independent experiments). *P* value was calculated with Fisher’s exact test. (**f**) 3D time-lapse imaging of MTOCs in oocytes expressing EGFP-Cep192 (MTOCs) and mCherry-BicD2-CT (not shown) treated with either DMSO (BicD2-CT) or monastrol (BicD2-CT+monastrol). Z-projection, 25 sections, every 1.5 μm. Representative examples from two independent experiments. (**g**) The mean number of MTOCs within the spindle region plotted over time. Error bars show s.d. Number of oocytes analysed for each condition is specified in brackets (aggregation over two independent experiments). (**h**) 3D time-lapse imaging of MTOCs in oocytes expressing EGFP-Cep192 (green, MTOCs), H2B-mRFP1 (magenta, chromosomes) and P150-CC1 (dynein inhibition) treated with DMSO (P150-CC1) or monastrol (P150-CC1+monastrol). Z-projection, 25 sections, every 1.5 μm. Scale bar, 10 μm. Time, h:min from NEBD. Representative examples from two independent experiments (>12 oocytes total for each condition). (**i**,**j**) Oocytes were scored for **i** MTOC fragmentation and **j** bipolar spindle formation. Number of oocytes analysed for each condition is specified in brackets (aggregation over two independent experiments). *P* values were calculated with Fisher’s exact test.

Next, we investigated the second phase of MTOC fragmentation, which took place after NEBD. In this phase, MTOCs were further fragmented into smaller MTOCs, which were subsequently separated from each other and distributed evenly between two spindle poles (Fig. 1; Supplementary Movies 1 and 2). By imaging MTOCs with high temporal resolution, we were able to track individual fragmentation events occurring after NEBD. This revealed MTOC fragments rapidly separating from each other (Fig. 3a, arrowheads; Supplementary Movie 2). A likely candidate responsible for post-NEBD MTOC fragmentation was KIF11, as this motor protein can drive centrosome separation after NEBD^26^. We first analysed KIF11 localization during MTOC fragmentation. KIF11 was absent from MTOCs before NEBD (Fig. 3b, arrowheads), which is consistent with our observation that KIF11 is dispensable for MTOC stretching along the nuclear envelope. Consistent with a role in the second phase of MTOC fragmentation, KIF11 was rapidly recruited to microtubules post NEBD and became enriched at the spindle poles during spindle assembly (Fig. 3b,c). To test directly whether post-NEBD MTOC fragmentation requires KIF11, we treated oocytes with monastrol. Consistent with our earlier results, KIF11 inhibition did not affect MTOC fragmentation before NEBD (Fig. 3d, time 0:00). However, MTOC fragmentation stopped immediately after NEBD. Instead, the MTOCs rapidly merged back together to form a single large MTOC at the centre of a monoaster (Fig. 3d,e). To quantify the contribution of KIF11 specifically to post-NEBD MTOC fragmentation, we prevented MTOC fragmentation before NEBD by expressing BicD2-CT. In oocytes expressing BicD2-CT alone, MTOC fragmentation initiated after NEBD, resulting in a sharp increase in MTOC number (Fig. 3f,g). However, addition of monastrol to BicD2-CT-expressing oocytes completely blocked post-NEBD MTOC fragmentation (Fig. 3f,g). The failure to fragment MTOCs after NEBD on KIF11 inhibition could represent a direct role for KIF11 in MTOC fragmentation, or an indirect consequence of monopolar spindle formation. To address this, we allowed bipolar spindles to assemble in the absence of KIF11 activity by simultaneous inhibition of KIF11 and dynein^27^ (Supplementary Fig. 6). MTOCs failed to fully fragment and split between the two spindle poles in 85% of bipolar spindles that assembled without KIF11 activity (Fig. 3h–j; Supplementary Fig. 6). Together, these results demonstrate that KIF11 promotes MTOC fragmentation after NEBD. Fragmented MTOCs can then be sorted between spindle poles by Hurp-mediated reorganization of spindle microtubules^28^.

### PLK1-dependent decondensation of MTOC structure

**Figure 4:**
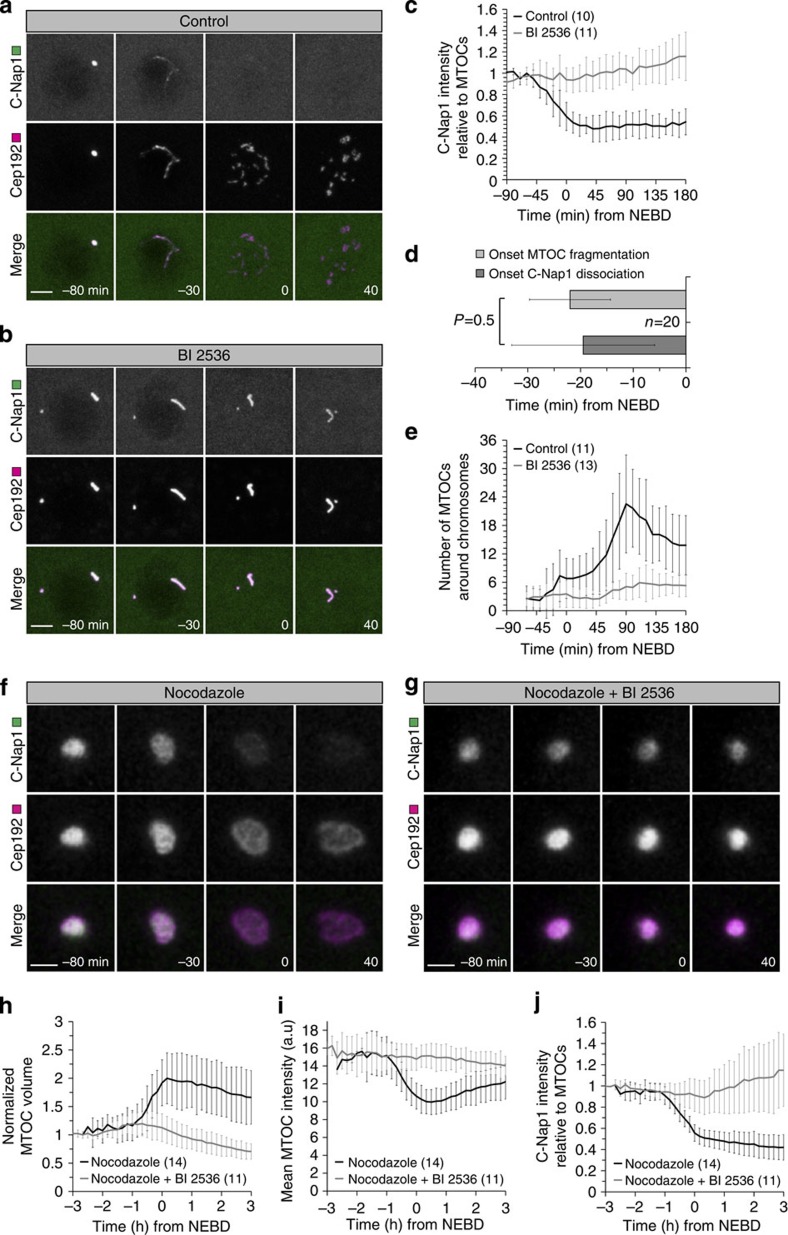
PLK1-dependent decondensation of MTOC structure facilitates MTOC fragmentation. (**a**,**b**) 3D time-lapse imaging of MTOCs in live mouse oocytes expressing mEGFP-C-Nap1 (green, C-Nap1) and mCherry-Cep192 (magenta, MTOCs) treated with (**a**) DMSO (control) or (**b**) BI 2536. Z-projection, 25 sections, every 1.5 μm. Scale bar, 10 μm. Time, minutes from NEBD. Representative examples from two independent experiments (>10 oocytes total for each condition). (**c**) The ratio of the mean C-Nap1 intensity to the mean Cep192 intensity within the MTOC region was normalized to the initial ratio and plotted over time. Error bars show s.d. Number of oocytes analysed for each condition is specified in brackets (aggregation over two independent experiments). (**d**) Timing of onset of MTOC fragmentation (when MTOCs first begin to stretch) and onset of C-Nap1 dissociation (when C-Nap1/Cep192 intensity ratio fell below 0.75) were quantified from oocytes expressing mEGFP-C-Nap1 and mCherry-Cep192. 20 oocytes from four independent experiments were analysed. Error bars show s.d. *P* value was calculated with Student’s *t*-test. (**e**) The mean number of MTOCs surrounding chromosomes was quantified from oocytes expressing EGFP-Cep192 (MTOCs) and H2B-mRFP1 (chromosomes) treated with either DMSO (control) or BI 2536. Error bars show s.d. Number of oocytes analysed for each condition is specified in brackets (aggregation over two independent experiments). (**f**,**g**) 3D time-lapse imaging of MTOCs from live mouse oocytes expressing mEGFP-C-Nap1 (green, C-Nap1) and mCherry-Cep192 (magenta, MTOCs) treated with (**f**) nocodazole or (**g**) nocodazole+BI 2536. Z-projection, 17 sections, every 1.5 μm. Scale bar, 5 μm. Time, minutes from NEBD. Representative examples from two independent experiments (>10 oocytes total for each condition). (**h**–**j**) Data sets shown in **f**,**g** were used to quantify (**h**) MTOC volume (normalized to initial value), (**i**) the mean MTOC intensity and (**j**) ratio of the mean C-Nap1 intensity to the mean Cep192 intensity within the MTOC region (normalized to initial ratio). Error bars show s.d. Number of oocytes analysed for each condition is specified in brackets (aggregation over two independent experiments).

Our results so far show that the sequential action of the two motor proteins, dynein (before NEBD) and KIF11 (after NEBD), drives MTOC fragmentation. Next, we investigated whether MTOC fragmentation is solely driven by the force of microtubule motor proteins or whether changes in MTOC structure facilitate their fragmentation. In mitosis, centrosomes are thought to be connected with each other through a subset of centrosomal proteins, which form a linker between the two mother centrioles. These centrosomal linker proteins dissociate at mitotic entry, which is thought to initiate centrosome separation^29^. Consistent with previous findings^30^, we found that the centrosomal linker protein C-Nap1 localized to unfragmented acentriolar MTOCs at the onset of oocyte maturation (Fig. 4a, time −80 min). This suggests that C-Nap1 binds to pericentriolar material independently of centrioles. Remarkably, we found that C-Nap1 dissociated from MTOCs as they fragmented (Fig. 4a,c), and the onset of C-Nap1 dissociation and MTOC fragmentation occurred with similar timing (Fig. 4d).

In mitosis, both C-Nap1 dissociation and centrosome separation are initiated by polo-like kinase PLK1 (ref. 31). To test whether PLK1 drives MTOC fragmentation, we blocked PLK1 activity with the small molecular inhibitor BI 2536 (ref. 32). Indeed, we found that MTOCs remained largely intact when PLK1 was inhibited (Fig. 4b,e), and C-Nap1 remained associated with MTOCs (Fig. 4b,c). This defect in MTOC fragmentation was not due to an inability to recruit or activate the microtubule motor proteins dynein and KIF11 that we identified to drive MTOC fragmentation (Supplementary Fig. 7). We therefore hypothesized that PLK1 is required to modify the structure of MTOCs so that they can be fragmented. To test this hypothesis, we imaged MTOCs with or without BI 2536 in the presence of nocodazole. In this experiment, microtubules are absent (Supplementary Fig. 8) and therefore any contribution from microtubules and motor proteins to MTOC morphology is eliminated, allowing us to specifically study changes in MTOC structure. Strikingly, we found that there was a PLK1-dependent decondensation of MTOC structure around the time when MTOC fragmentation would normally occur (Fig. 4f–j; Supplementary Movie 4). In control oocytes (nocodazole), the volume of MTOCs increased and their density decreased (Fig. 4f,h,i), coinciding with the dissociation of C-Nap1 from MTOCs (Fig. 4f,j). However, when PLK1 was inhibited (nocodazole+BI 2536), MTOC decondensation was completely blocked: MTOCs remained in a condensed state with C-Nap1-associated (Fig. 4g–j). MTOC decondensation was unaffected by persistent and increased levels of C-Nap1 on MTOCs (Supplementary Fig. 9), suggesting that C-Nap1 dissociation *per se* is not required for this process. Together, these results show that PLK1 is essential for the decondensation of the MTOC structure and MTOC fragmentation.

### MTOC fragmentation facilitates bipolar spindle assembly

We next investigated the function of MTOC fragmentation in meiotic spindle assembly. If MTOC fragmentation plays a role in regulating spindle bipolarity, disrupting MTOC fragmentation should perturb spindle assembly. We first tested the role of pre-NEBD MTOC fragmentation in spindle assembly by expressing BicD2-CT to block MTOC stretching on the nuclear envelope. BicD2-CT expression caused an abnormal distribution of microtubules immediately after NEBD: instead of being homogeneously distributed throughout the volume of the former nucleus, microtubules were nucleated only locally, in the area of the unfragmented MTOCs (Supplementary Fig. 10a–c). Nonetheless, BicD2-CT-expressing oocytes quickly recovered and assembled normal bipolar spindles with timing similar to control oocytes (Supplementary Fig. 10a,d–g). Therefore, pre-NEBD MTOC fragmentation is not essential for bipolar spindle assembly, but may facilitate the spindle assembly process by ensuring that all chromosomes are rapidly in contact with microtubules on NEBD. These data also show that KIF11-dependent MTOC fragmentation after NEBD is sufficient to drive bipolar spindle assembly.

**Figure 5:**
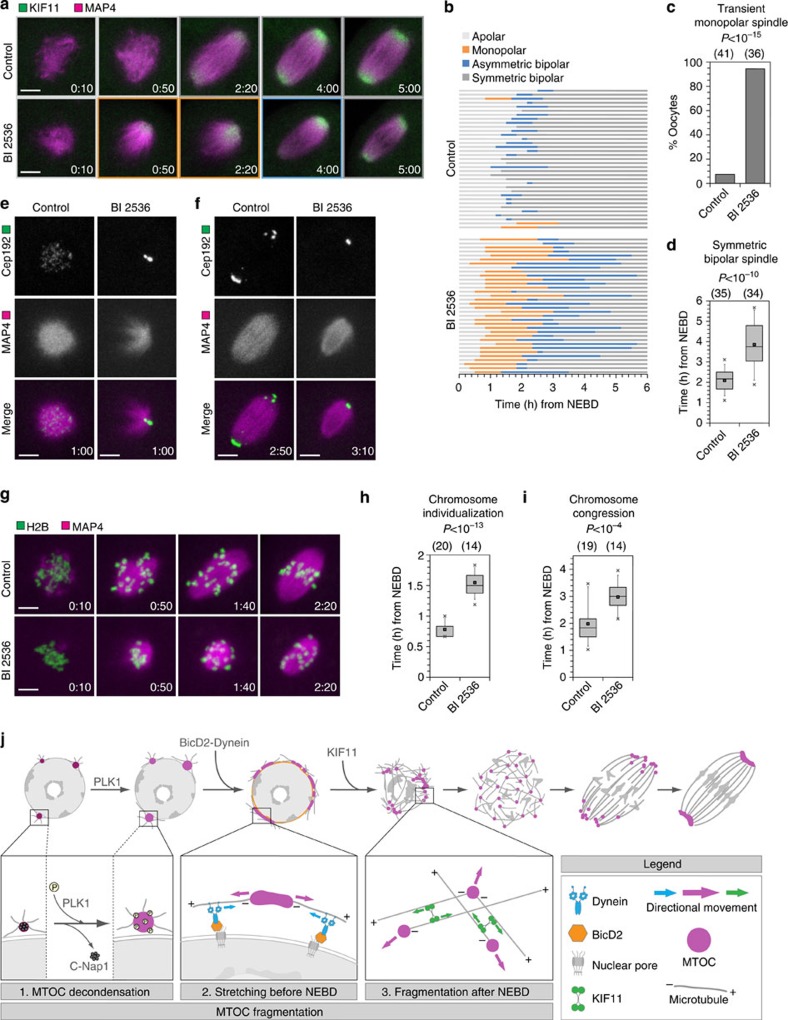
PLK1 activity is required for timely spindle assembly and chromosome congression. (**a**) 3D time-lapse imaging of DMSO- (control) or BI 2536-treated oocytes expressing KIF11-mEGFP (green, spindle poles) and mCherry-MAP4 (magenta, microtubules). Z-projection, seven sections, every 4 μm. Scale bar, 10 μm. Time, h:min from NEBD. Representative examples from two independent experiments (>20 oocytes for each condition). Coloured boxes correspond to stages of spindle assembly illustrated in **b**. (**b**) Stages of spindle assembly quantified from data sets shown in **a** and also from oocytes expressing KIF11-mEGFP and H2B-mRFP1 (not shown) are plotted for individual oocytes (each bar) over time. Presence of a spindle pole was determined as a focused accumulation of KIF11-mEGFP signal. Aggregation over five independent experiments. (**c**) Oocytes from **b** were scored for presence of a transient monopolar spindle. Number of oocytes analysed for each condition is specified in brackets above each column. *P* value was calculated with Student’s *t*-test. (**d**) Box plots showing time of symmetric bipolar spindle formation quantified from oocytes in **b**. Number of oocytes analysed for each condition is specified in brackets*. P* values were calculated with Student’s *t*-test. (**e**,**f**) Images from oocytes expressing EGFP-Cep192 (green, MTOCs) and mCherry-MAP4 (magenta, microtubules) treated with DMSO (control) or BI 25136. Z-projection, 25 sections, every 1.5 μm. Scale bar, 10 μm. Time, h:min from NEBD. Representative examples from two independent experiments (12 oocytes for each condition). (**g**) 3D time-lapse imaging of oocytes expressing H2B-mEGFP (green, chromosomes) and mCherry-MAP4 (magenta, microtubules) treated with DMSO (control) or BI 2536. Z-projection, 25 sections, every 1.5 μm. Scale bar, 10 μm. Time, h:min from NEBD. Representative examples from two independent experiments (12 oocytes for each condition). (**h**,**i**) Box plots showing time of (**h**) chromosome individualization and (**i**) chromosome congression quantified from oocytes in **g** and also from oocytes expressing KIF11-mEGFP and H2B-mRFP1 (not shown). Number of oocytes analysed for each condition is specified in brackets (aggregation over five independent experiments)*. P* values were calculated with Student’s *t*-test. (**j**) A model for bipolar spindle assembly in mouse oocytes showing three-step mechanism of MTOC fragmentation.

To address the role of MTOC fragmentation in spindle assembly, we inhibited PLK1, which we found to completely inhibit the fragmentation of MTOCs (Fig. 4). To analyse potential defects in spindle assembly after PLK1 inhibition, we imaged microtubules together with KIF11 as a reporter for the spindle poles (Fig. 5a). In control oocytes, spindle assembly followed a typical pattern: first, MTOCs nucleated a microtubule mass without well-defined poles (Fig. 5a,b, light grey boxes and bars); second, microtubules were progressively focused to form two equal spindle poles and the spindle elongated (Fig. 5a,b, dark grey boxes and bars). However, bipolar spindle assembly was severely compromised when MTOC fragmentation was blocked by inhibiting PLK1 (Supplementary Movie 5). In contrast to control oocytes, 95% of PLK1-inhibited oocytes assembled transient monopolar spindles (Fig. 5a,b, orange boxes and bars; Fig. 5c). Transient monopolar spindles were also assembled in PLK1-depleted oocytes, and this phenotype could be rescued by expression of human PLK1 (Supplementary Fig. 11), thus confirming the specificity of BI 2536 treatment for PLK1 inhibition in mouse oocytes. These monopolar spindles were nucleated from unfragmented MTOCs residing at the monopole (Fig. 5e; Supplementary Fig. 12b; Supplementary Movie 6). PLK1 inhibition did not significantly reduce the amount of microtubules at MTOCs (Supplementary Fig. 12a–c); however, the total amount of microtubules present during spindle assembly were reduced (Supplementary Fig. 12d). It is unlikely that the reduction in microtubules on PLK1 inhibition contributes to the formation of monopolar spindles because reducing the number of microtubules by other means, for instance, by inhibiting the small GTPase Ran or by low doses of nocodazole, does not lead to monopolar spindles^7^,^33^. Strikingly, in the rare occasions that MTOC fragmentation was delayed well beyond NEBD in control oocytes, transient monopolar spindles identical to those seen on PLK1 inhibition were also assembled (Supplementary Movie 7). Altogether, these data suggest that the transient monopolar spindles seen following PLK1 inhibition are due to a failure in MTOC fragmentation. However, we cannot exclude the possibility that PLK1 additionally regulates bipolar spindle assembly through processes other than MTOC fragmentation. Following the prolonged monopolar spindle phase, the monopole split unequally and a second, smaller spindle pole formed (Fig. 5a,b blue boxes and bars; Supplementary Movies 5 and 6). The asymmetric spindles that were formed in this way persisted for an average of 1.6±1.1 h (Fig. 5a,b, blue boxes and bars). Such asymmetric spindles were also transiently seen on spindle bipolarization in control oocytes (Fig. 5b, blue bars). However, asymmetric spindles in control oocytes quickly equilibrated, possibly driven by the rapid redistribution of fragmented MTOCs (Fig. 1a)^28^. Indeed, MTOCs were evenly distributed between both poles in the symmetric spindles in control oocytes, but unevenly distributed in the asymmetric spindles caused by PLK1 inhibition (Fig. 5f). The weaker spindle pole sometimes even lacked any detectable MTOCs, indicative of a failure in MTOC fragmentation (Fig. 5f; Supplementary Movie 6). PLK1-inhibited oocytes eventually formed symmetric bipolar spindles (Fig. 5a,b, dark grey boxes and bars); however, the formation of symmetric spindles was significantly delayed compared with control oocytes (Fig. 5d). A similar delay in bipolar spindle assembly was also observed in PLK1-depleted oocytes (Supplementary Fig. 11h,i). Altogether, these results indicate that the PLK1-driven fragmentation of MTOCs promotes the timely assembly of a symmetric bipolar spindle and prevents the formation of monopolar and asymmetric spindles.

We next investigated whether the formation of monopolar and asymmetric spindle intermediates on PLK1 inhibition had any consequences for chromosomes in the spindle. Because PLK1 is required to establish kinetochore–microtubule attachments^32^, which are essential for chromosome alignment and segregation, we analysed earlier events, namely chromosome individualization and chromosome congression, which do not rely on kinetochore–microtubule attachments^34^. In mouse oocytes, chromosomes are initially clustered around the nucleolus and in the nuclear periphery. Thus, they must be individualized on NEBD. In control oocytes, chromosomes were individualized within less than an hour after NEBD (Fig. 5g,h, time 0:50). However, when PLK1 was inhibited, chromosome individualization was significantly delayed (Fig. 5g,h). Instead, chromosomes remained clumped together towards the ends of microtubules emanating from a monopole (Fig. 5g, time 0:50). This indicates that the formation of an apolar microtubule mass, which depends on MTOC fragmentation, is necessary to ensure the timely individualization of chromosomes following NEBD. Next, we investigated whether chromosome congression was affected when MTOC fragmentation was blocked. In control oocytes, chromosomes began to congress into a prometaphase belt ∼2 h after NEBD (Fig. 5g,i). Inhibition of PLK1 significantly delayed chromosome congression (Fig. 5g,i). This is likely to be caused by the delay in assembling a symmetric spindle in these oocytes, although we cannot exclude the possibility that PLK1 plays a more direct role in chromosome congression. Together, these data show that inhibition of MTOC fragmentation leads to transient monopolar and asymmetric spindles, which delays chromosome individualization and congression in oocytes.

## Discussion

In summary, mouse oocytes assemble a bipolar spindle by fragmenting their acentriolar MTOCs into a high number of small MTOCs to be able to then regroup and merge them into two equal spindle poles. This fragmentation of MTOCs is essential for timely assembly of a symmetric bipolar spindle. We show that MTOC fragmentation is achieved in a three-step process that is driven by PLK1 (Fig. 5j). In the first step, PLK1 triggers a decondensation of MTOC structure and the release of the centrosomal linker protein C-Nap1. In the second step, BicD2 recruits dynein to the nuclear envelope, which stretches the MTOCs into ribbons and starts to fragment them. In the third step, KIF11 further fragments the MTOCs so that they can be evenly distributed between two the spindle poles. Blocking MTOC fragmentation leads to defects in spindle assembly, which delay chromosome individualization and congression. It is crucial that oocytes prevent such delays because all chromosomes need to be correctly attached to the spindle by the time the oocyte progresses into anaphase. This is particularly important in oocytes as it takes several hours to correct erroneous kinetochore–microtubule attachments^35^, and defects in this process are often missed by the spindle assembly checkpoint, which can lead to the production of an aneuploidy egg^36^.

In addition to revealing how oocytes assemble a bipolar spindle from multiple MTOCs, this study also provides insights into centrosome separation in mitotic cells. Our data show that PLK1 facilitates MTOC fragmentation by triggering the decondensation of the structure of acentriolar MTOCs. PLK1 has also been reported to drive the separation of centrosomes in mitosis^31^. Our data raise the interesting possibility that PLK1 facilitates centrosome separation by decondensation of the pericentriolar material. An important challenge for the future will be to identify the substrate(s) of PLK1 responsible for regulating MTOC structure. Although C-Nap1 and other centrosomal linker proteins dissociate during centrosome separation and MTOC fragmentation, it is unclear whether their dissociation is of functional relevance to MTOC fragmentation and centrosome separation. Since MTOCs in mouse oocytes lack centrioles, C-Nap1 also cannot act as a centriolar linker. Its dissociation from MTOCs might instead be indicative of a PLK1-induced change in the MTOC structure that makes MTOCs nonpermissive for C-Nap1 binding and facilitates MTOC fragmentation in meiosis and centrosome separation in mitosis.

This study also provides new insights into the functional organization of MTOCs. We show that MTOCs exhibit remarkable plasticity: MTOCs can alter their density, be stretched into ribbon-like structures, fragment into many smaller functional units and also merge, all to facilitate different stages of spindle assembly in the oocyte. Interestingly, centrosome fragmentation has also been observed during anaphase of mitosis, which may facilitate the re-establishment of an interphase microtubule array^37^. Thus, MTOC plasticity may be a general mechanism to rapidly adapt MTOC function to different cellular requirements.

## Methods

### Preparation and culture of oocytes

All mice were maintained in a specific pathogen-free environment according to UK Home Office regulations. Oocytes were isolated from ovaries of 8-week-old FVBn female mice and cultured in M2 medium covered by mineral oil at 37 °C. Only large oocytes with a centrally located nucleus were used, as these are more likely to be meiotically competent. For experiments where chromosomes were imaged, only those oocytes that had a surround-nucleolar chromosome configuration were used. Isolated oocytes were maintained in a prophase arrest by addition of 250 μm dbcAMP, microinjected with mRNA as described previously^7^ and then released into a dbcAMP-free medium once proteins were expressed, typically 3 h after microinjection. In some experiments, oocytes were treated with 1 μM nocodazole (Sigma), 100 μM monastrol (Sigma), 250 nM BI 2536 (Selleckchem) or corresponding amounts of dimethylsulphoxide (DMSO) in controls. We initially tried a range of concentrations of BI 2536 from 100 nM to 1 μM and saw the same phenotypes at all concentrations, which were also observed by PLK1 knockdown with short interfering RNA (siRNAs; Supplementary Fig. 11). We chose to use 250 nM BI 2536 because it is close to the concentrations previously used in cultured mitotic cells (100 nM^32^)) and in mouse embryos (500 nM (ref. 38)). For knockdown of PLK1 using RNA interference (RNAi), a mix of the following siRNAs were used: 5′- CCUCUCAAAGUCCUCAAUATT -3′ and 5′- CCACUGUAUGAAUUGUAUATT -3′. For control, a scrambled negative control siRNA was used (Qiagen SI03650318). siRNAs were injected to a final concentration of 30 nM into follicle-enclosed oocytes from 11-day-old (C57BL × CBA) F1 females and then cultured for 9–10 days as described in ref. 39. Oocytes were then isolated from the follicles and cultured as described above. For live imaging of RNAi oocytes, 3D time-lapse images were acquired using Zeiss’s MultiTime Series macro. The entire oocyte (∼90 μm × 90 μm) in the *x–y* dimension, and a region of 24 μm (five confocal *z*-sections every 6.0 μm) surrounding the H2B-mRFP1 or mCherry-MAP4 signal in the *z*-dimension, was imaged every 12 min for a total of ∼12 h following release from dbcAMP, covering NEBD to MII. Under these conditions, 92% (*n*=51) of ctrl siRNA oocytes extruded a polar body.

### RT–qPCR

mRNA was extracted from oocytes using the RNeasy Micro Kit (Qiagen) and cDNA was generated using the High-Capacity RNA-cDNA Kit (Applied Biosystems). Real-time PCR was performed with the 7900HT Fast Real-Time PCR System (Applied Biosystems) using SYBR green. *Gapdh* mRNA was used for normalization. The following primer sets were used: *Gapdh*, forward 5′- AGAGCTGAACGGGAAGCTCACT -3′, reverse 5′- TGCCTGCTTCACCACCTTCTTGAT -3′; *Plk1*, forward 5′- TGTAGTTTTGGAGCTCTGTCG -3′, reverse 5′- TCCCTGTGAATGACCTGATTG -3′.

### Expression constructs and mRNA synthesis

To generate the constructs for *in vitro* mRNA synthesis, we fused the previously published coding sequences with mEGFP (Clontech), mCherry^40^ or tdEos^14^ to obtain tdEos-Cep192 (ref. 12), mCherry-BicD2 and mCherry-BicD2-CT^25^, KIF11-mEGFP^41^, C-Nap1-mEGFP^42^, mCherry-MAP4 (ref. 43), mEGFP-H2B^44^, EB3-mCherry^45^, mEGFP-Pericentrin (PACT domain)^46^, mEGFP-α-Tubulin^47^ and mEGFP-PLK1 (ref. 48) and inserted these into pGEMHE for *in vitro* transcription. The Nek2-K37R construct was generated by inserting the Nek2-coding sequence^49^ into pGEMHE followed by site-directed mutagenesis (Quikchange, Agilent) using the following primers: sense 5′- TGATGGCAAGATATTAGTTTGGAgAGAACTTGACTATGGCTC -3′; antisense 5′- GAGCCATAGTCAAGTTCTcTCCAAACTAATATCTTGCCATCA -3′. These constructs as well as pCR3.1-EGFP-Cep192 and pCR3.1-mCherry-Cep192 (ref. 12), pET28-P150-CC1-His^50^ and pGEMHE-H2B-mRFP1 (ref. 7) were linearized, and capped mRNA was synthesized with T7 polymerase (Ambion mMessage mMachine kit, according to the manufacturer’s instructions).

### Confocal microscopy

Images were acquired with a Zeiss LSM710 or a Zeiss LSM780 microscope. Microscopes were equipped with a Zeiss environmental incubator box, which for live imaging maintained oocytes at 37 °C without CO_2_. Oocytes were imaged with a × 40 C-Apochromat 1.2 NA water-immersion objective for live oocytes and a × 63 C-Apochromat 1.2 NA water-immersion objective for fixed oocytes as described previously^7^. In some images, shot noise was decreased with a Gaussian filter.

### Immunofluorescence microscopy

Oocytes were fixed for 30–60 min at 37 °C in 100 mM HEPES (pH 7; titrated with KOH), 50 mM EGTA (pH 7; titrated with KOH), 2% formaldehyde (methanol-free) and 0.2% Triton X-100, based on previously published methods^51^. Fixed oocytes were incubated in PBS with 0.1% Triton X-100 overnight at 4 °C. Antibody incubations were performed in PBS, 3% BSA and 0.1% Triton X-100. Primary antibodies used were mouse anti-pericentrin (30, BD Biosciences 611815; 1:750), mouse anti-γ-tubulin (GTU88, Sigma T6557; 1:3,000), rat anti-tyrosinated-α-tubulin (YOL1/34, AbD Serotec MCA78G; 1:3,000) and mouse anti-PLK1 (35–206, Abcam Ab17056; 1:1,000). Secondary antibodies used were Alexa-Fluor-488-labelled anti-mouse (Molecular Probes A11029; 1:400) and Alexa-Fluor-647-labelled anti-rat (Molecular Probes A21247; 1:400). DNA was stained with 5 mg ml^−1^ Hoechst 33342 (Molecular Probes H3570).

### 3D reconstruction of MTOCs

To label MTOCs, oocytes were microinjected with mRNAs encoding fluorescently labelled Cep192. 3D time-lapse images were acquired using Zeiss’s MultiTime Series macro. The entire oocyte (∼90 μm × 90 μm) in the *x–y* dimension, and a region of 37.5 μm (25 confocal *z*-sections every 1.5 μm) surrounding the H2B-mRFP1 signal in the *z*-dimension, was imaged every 10 min for a total of ∼12 h following release from dbcAMP, covering NEBD to MII. Under these conditions, 88% (*n*=90) of oocytes extruded a polar body, compared with 91% (*n*=109) of nonimaged oocytes. We segmented the MTOCs by applying a threshold using the isosurface function of Imaris (Bitplane) and manually removed MTOCs that were not incorporated into the spindle region. The threshold value used for 3D reconstruction of MTOCs was determined on an oocyte-to-oocyte basis depending on the MTOC signal to noise within each oocyte. Cep192 was chosen over other MTOC markers such as Pericentrin because of the high MTOC signal-to-noise ratio (Supplementary Fig. 1c), allowing small MTOCs to be visualized. Values for the number and volume of MTOCs were exported into Microsoft Excel.

### Quantification of MTOC stretching

Oocytes were microinjected and imaged as described for 3D reconstruction of MTOCs. Values for MTOC sphericity were calculated as the ratio of the surface area of a sphere (with the same volume as the given MTOC) to the surface area of the MTOC. The speed of MTOC stretching was calculated by measuring the change in length of MTOCs in the direction of MTOC stretching over time and divided by two, to obtain the speed of MTOC elongation at the leading edge.

### Fluorescence intensity measurements

To measure C-Nap1 intensity relative to MTOCs, oocytes expressing mEGFP-C-Nap1 and mCherry-Cep192 were imaged (25 *z*-confocal sections every 1.5 μm, 10-min intervals). MTOCs were segmented by applying a threshold in the Cep192 channel using the isosurface function of Imaris. The mean intensities for mEGFP-C-Nap1 and mCherry-Cep192 within the MTOC region were measured using Imaris and exported into Microsoft Excel. For background subtraction, the intensity in an area outside the oocyte was measured. The ratio of the mean C-Nap1 intensity to the mean Cep192 intensity was calculated and normalized to the initial value. To measure KIF11 intensity, oocytes expressing mEGFP-KIF11 and mCherry-MAP4 were imaged (seven *z*-confocal sections every 4 μm, 10-min intervals). The microtubules were segmented by applying a threshold in the mCherry-MAP4 channel using the isosurface function of Imaris. The mean intensity of KIF11-mEGFP in the microtubule region was measured, background-subtracted and normalized to the initial value. To measure total microtubule intensities, oocytes expressing mCherry-MAP4 or EB3-mCherry were imaged and the microtubule signal segmented by applying a threshold in the mCherry-MAP4 or EB3-mCherry channels using the isosurface function of Imaris. Total intensities (mean intensity × volume) were measured using Imaris and exported into Microsoft Excel.

### Statistics

Average (mean), s.d. and statistical significance based on Student’s *t*-test (two-tailed) for absolute values or Fisher’s exact test (two-tailed) for category values were calculated in Microsoft Excel. All box plots show the median (line), mean (small square), 1st, 99th (crosses), 5th, 95th (whiskers) and 25th and 75th percentiles (boxes).

## Additional information

**How to cite this article:** Clift, D. and Schuh, M. A three-step MTOC fragmentation mechanism facilitates bipolar spindle assembly in mouse oocytes. *Nat. Commun.* 6:7217 doi: 10.1038/ncomms8217 (2015).

## Supplementary Material

Supplementary Information Supplementary Figures 1-12.

Supplementary Movie 1 MTOCs fragment and merge during meiotic spindle assembly. (Left) 3D time-lapse imaging of MTOCs from an oocyte expressing EGFPCep192 (green, MTOCs) and H2B-mRFP1 (magenta, chromosomes). Zprojection, 25 sections, every 1.5 μm. (Right) 3D reconstruction of MTOCs from same oocyte. Time, h:min from NEBD. Same oocyte as shown in Fig. 1a,b.

Supplementary Movie 2 MTOCs fragment in two phases. 3D time-lapse imaging of MTOCs from an oocyte expressing EGFP-Cep192 (green, MTOCs) and H2B-mRFP1 (magenta, chromosomes). Z-projection, 25 sections, every 1.5 μm. Time, h:min from NEBD. Supplementary movie 3 MTOC stretching coincid

Supplementary Movie 3 MTOC stretching coincides with BicD2 recruitment to the nuclear envelope and requires BicD2-dynein interaction. 3D time-lapse imaging of oocytes expressing EGFP-Cep192 (green, MTOCs) and mCherry-BicD2 (magenta, left) or mCherry-BicD2-CT (magenta, right). Zprojection, 25 sections, every 1.5 μm. Time, minutes from NEBD. Same oocytes as shown in Fig. 2g,h.

Supplementary Movie 4 PLK1-dependenent decondensation of MTOC structure. 3D time-lapse imaging of MTOCs from oocytes expressing EGFP-Cep192 treated with nocodazole or nocodazole + BI 2536. Z-projection, 17 sections, every 1.5 μm. Time, h:min from NEBD. Same oocytes as shown in Fig. 4f,g.

Supplementary Movie 5 PLK1 inhibition causes transient monopolar spindles. 3D Time-lapse imaging in control and BI 2536 treated live mouse oocytes expressing KIF11-mEGFP (green, spindle poles) and mCherry-MAP4 (magenta, microtubules). Z-projection, 7 sections, every 4 μm. Time, h:min from NEBD. Same oocytes as shown in Fig. 5a.

Supplementary Movie 6 MTOC dynamics and spindle assembly upon PLK1 inhibition D Time-lapse imaging of a BI 2536-treated live mouse oocyte expressing EGFPCep192 (green, MTOCs) and mCherry-MAP4 (magenta, microtubules). Zprojection, 25 sections, every 1.5 μm. Time, h:min from NEBD

Supplementary Movie 7 Delayed MTOC fragmentation in control oocytes causes transient monopolar spindles 3D Time-lapse imaging of a live mouse oocyte expressing EGFP-Cep192 (green, MTOCs) and mCherry-MAP4 (magenta, microtubules). Z-projection, 25 sections, every 1.5 μm. Time, h:min from NEBD.
