# Supplementary material for: A three-step MTOC fragmentation mechanism facilitates bipolar spindle assembly in mouse oocytes
Source: Nat Commun. 2015 Jul 6;6:7217. doi: 10.1038/ncomms8217 (PMC4501430; doi:10.1038/ncomms8217)
Supplement: Supplementary Information — Supplementary Figures 1-12. [file ncomms8217-s1.pdf]

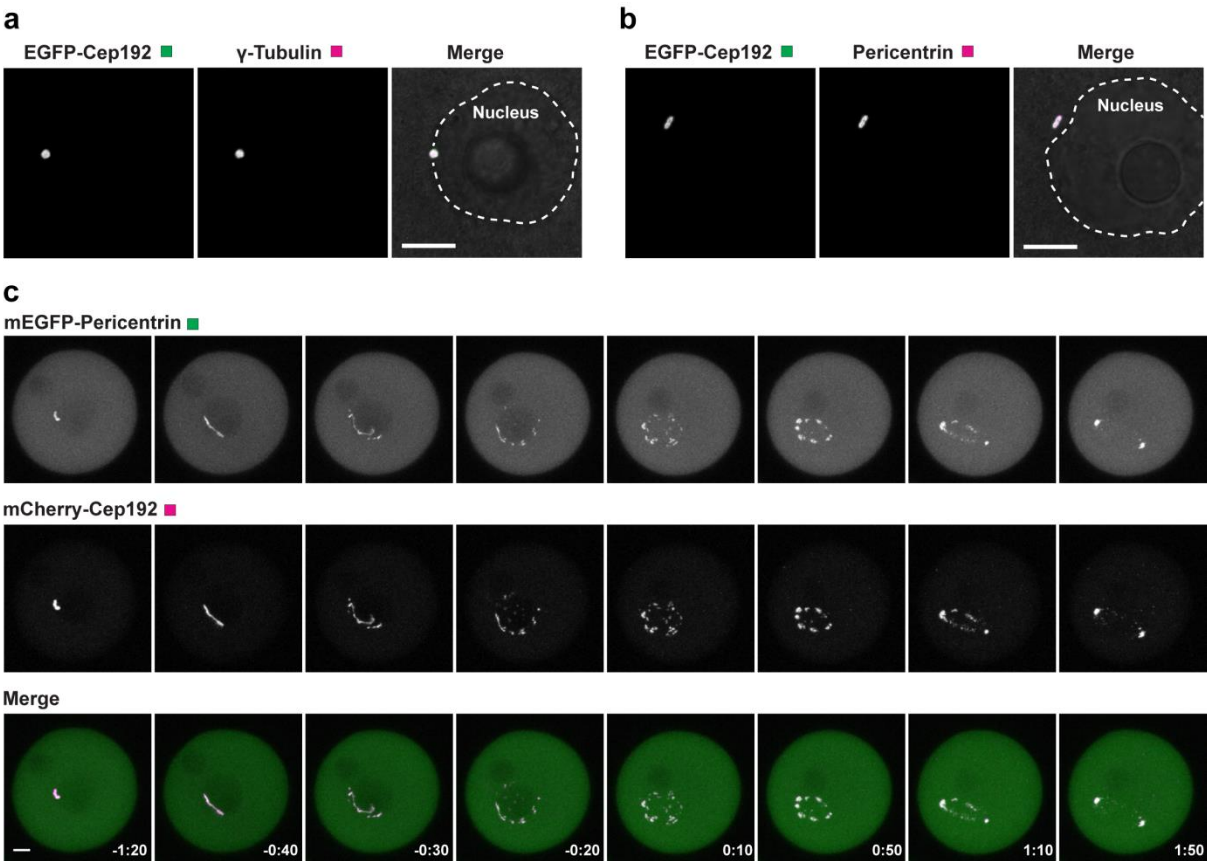

Supplementary Figure 1 Cep192 is a marker for MTOCs

(a,b) Oocytes expressing EGFP-Cep192 (green) were fixed in prophase and stained for **(a)** γ-tubulin (magenta) and **(b)** Pericentrin (magenta). Single confocal sections shown. Scale bar, 10 μm. **(c)** 3D Time-lapse imaging of MTOCs in live mouse oocytes expressing mEGFP-Pericentrin (green) and mCherry-Cep192 (magenta). Z-projection, 25 sections, every 1.5 μm. Scale bar, 10 μm. Time, minutes from NEBD. Representative examples from 2 independent experiments.

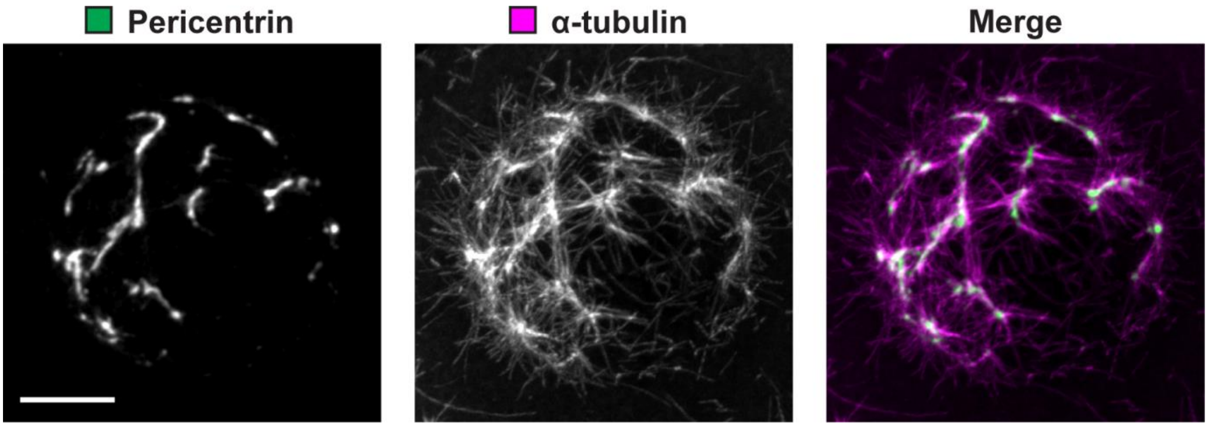

Supplementary Figure 2 Stretched MTOCs colocalize with microtubules on the nuclear envelope

Oocyte just prior to NEBD was fixed and stained for Pericentrin (green, MTOCs) and α-tubulin (magenta, microtubules). Z-projection, 44 sections, every 0.5 µm. Scale bar, 10 µm.

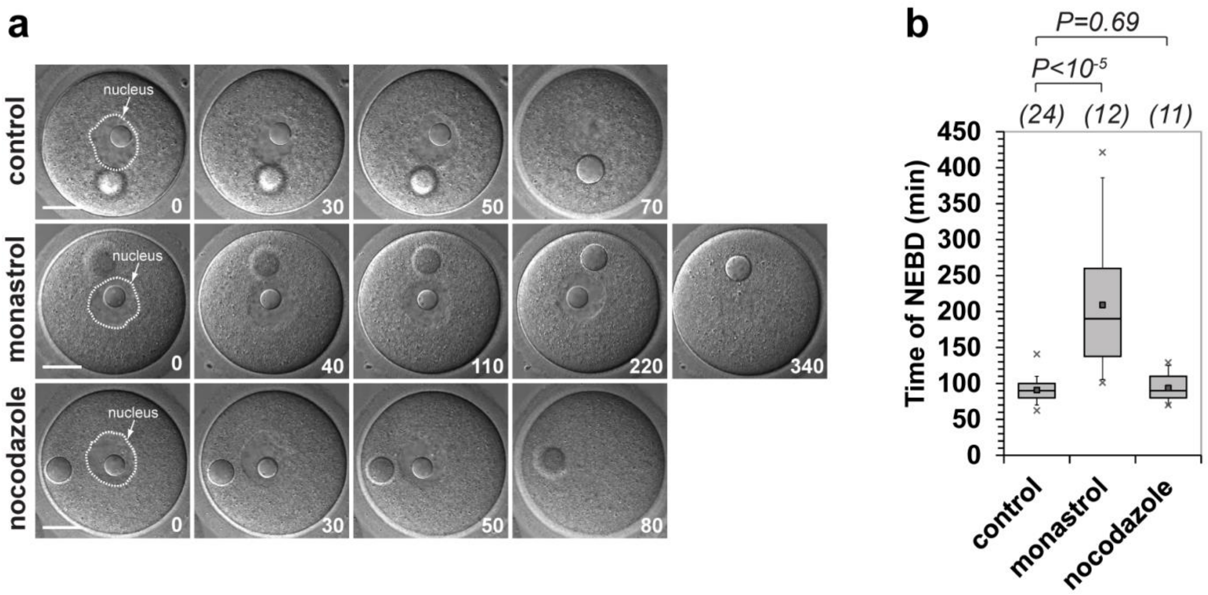

Supplementary Figure 3 Monastrol treatment delays onset of NEBD

(a) Differential interference constrast (DIC) time-lapse imaging of oocytes expressing EGFP-Cep192 and H2B-mRFP1 (not shown) treated with DMSO (control), monastrol or nocodazole. Dashed line outlines nucleus. Spherical structure in the cytoplasm is an oil droplet resulting from the microinjection procedure. Scale bar, 20 µm. Time, minutes from dbcAMP wash-out. **(b)** Box plots showing time of NEBD relative to dbcAMP wash-out quantified from oocytes shown in **a**. Number of oocytes analysed is specified in brackets above each box (aggregation over 2-4 independent experiments). *P* values were calculated with Student’s *t*-test.

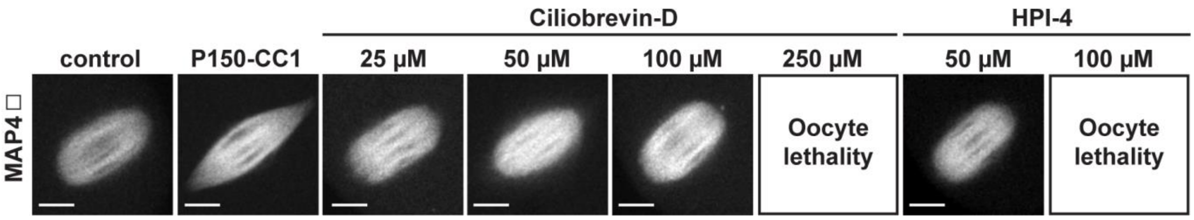

Supplementary Figure 4 P150-CC1 expression, but not Ciliobrevin-D treatment, inhibits dynein activity in mouse oocytes

Images of spindles from live mouse oocytes expressing mCherry-MAP4 (microtubules). Control oocytes have typical barrel-shaped spindle. Dynein inhibition (P150-CC1) causes distinctive diamond-shaped spindles. Treatment with Ciliobrevin-D or HPI-4 (Ciliobrevin analogue) does not affect spindle morphology.

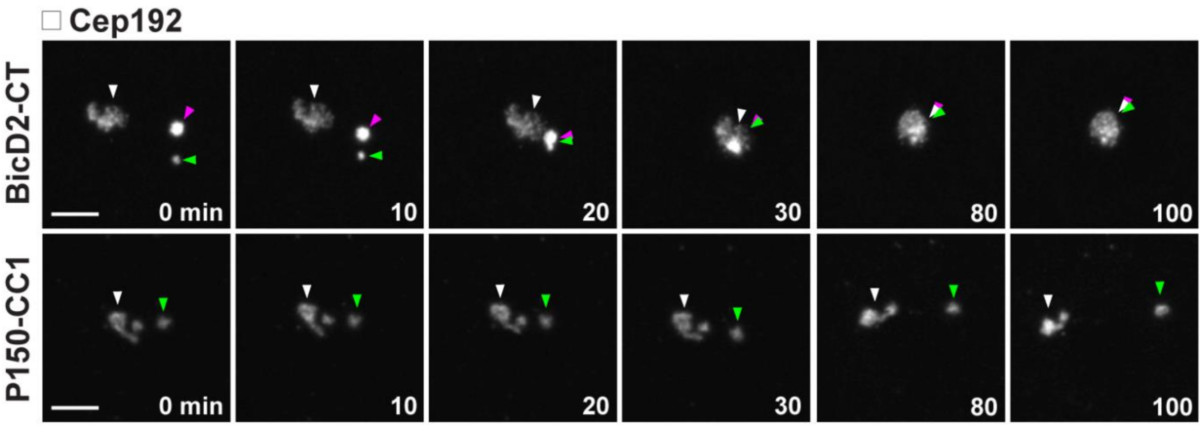

Supplementary Figure 5 Dynein-dependent merging of MTOCs is unaffected by BicD2-CT expression

3D Time-lapse imaging of MTOCs in live mouse oocytes expressing EGFP-Cep192 (MTOCs) and either mCherry-BicD2-CT (BicD2-CT) or P150-CC1 (P150-CC1) treated with monastrol. 25 sections, every 1.5 µm, every 10 minutes. Scale bar, 10 µm. Representative examples from 2 independent experiments (12 oocytes total for each condition). Arrowheads highlight movements of MTOCs. Global dynein inhibition (P150-CC1) inhibits MTOC merging after NEBD whilst inhibition of nuclear envelope dynein (BicD2-CT) has no affect.

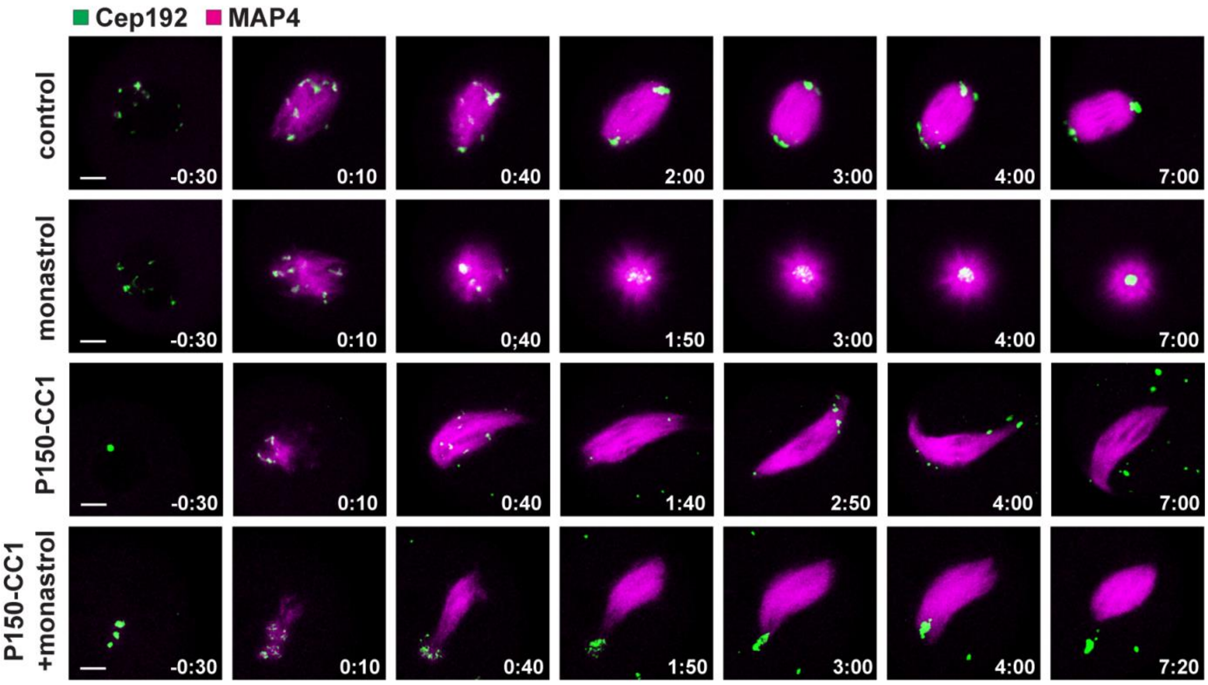

Supplementary Figure 6 Bipolar spindle formation in the absence of KIF11 activity

3D Time-lapse imaging of live mouse oocytes of the indicated condition expressing EGFP-Cep192 (green, MTOCs) and mCherry-MAP4 (magenta, microtubules). Z-projection, 7 sections, every 4 µm. Scale bar, 10 µm. Time, h:min from NEBD. Representative examples from 2 independent experiments (>12 oocytes total for each condition).

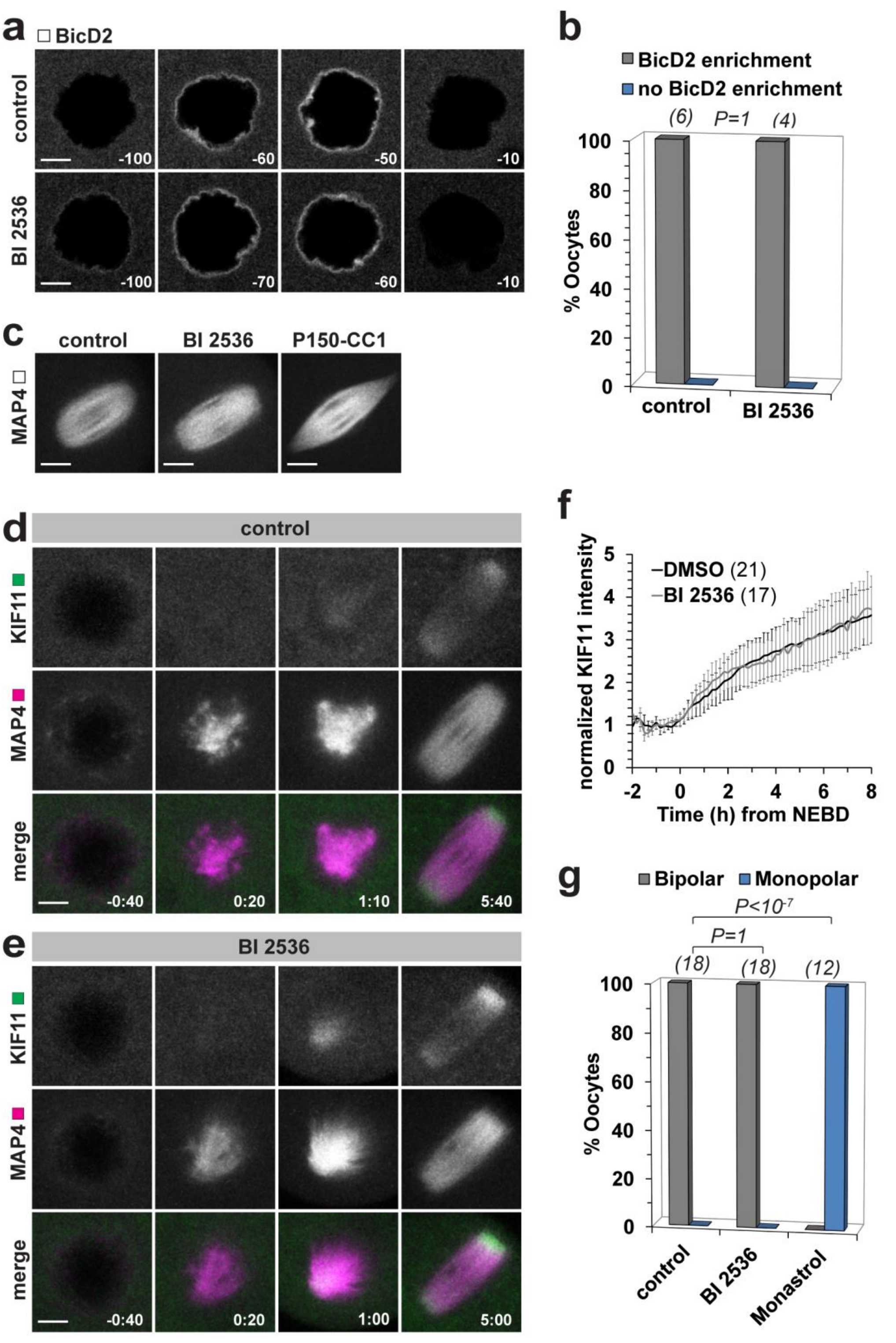

Supplementary Figure 7 PLK1 inhibition does not affect the localization or activity of BicD2-dynein and KIF11.

(a) 3D time-lapse imaging of live DMSO- (control) or BI 2536-treated mouse oocytes expressing mCherry-BicD2. Single z-sections from representative examples are shown.

Scale bar, 10 µm. Time, minutes from NEBD. **(b)** Data sets from **a** were scored for BicD2 enrichment at nuclear envelope. Number of oocytes analysed for each condition is specified in brackets above each column. *P* value was calculated with Fisher's exact test. **(c)** Images of spindles from live mouse oocytes expressing mCherry-MAP4 (microtubules). Control and BI 2536-treated oocytes have typical barrel-shaped spindle. Dynein inhibition (P150-CC1) causes distinctive diamond-shaped spindles. **(d,e)** 3D time-lapse imaging of oocytes expressing KIF11-mEGFP (green, KIF11) and mCherry-MAP4 (magenta, microtubules) treated with **(d)** DMSO (control) or **(e)** BI 2536. Z-projection, 7 sections, every 4 µm. Scale bar, 10 µm. Time, h:min from NEBD. Representative examples from 2 independent experiments (>16 oocytes total for each condition). **(f)** Mean KIF11-mEGFP intensity within microtubule (mCherry-MAP4) region, normalized to initial value, is plotted over time. Error bars show s.d.. Number of oocytes analysed for each condition is specified in brackets (aggregation over 2 independent experiments). **(g)** Oocytes expressing EGFP-Cep192 (MTOCs) and H2B-mRFP1 (chromosomes) were treated with DMSO (control), BI 2536 or monastrol and scored at 6 hours after NEBD for bipolar or monopolar spindles. Control and BI 2536-treated oocytes form bipolar spindles, whereas KIF11 inhibition causes monopolar spindles. Number of oocytes analysed for each condition is specified in brackets above each column (aggregation over 2-3 independent experiments). *P* values were calculated with Fisher's exact test.

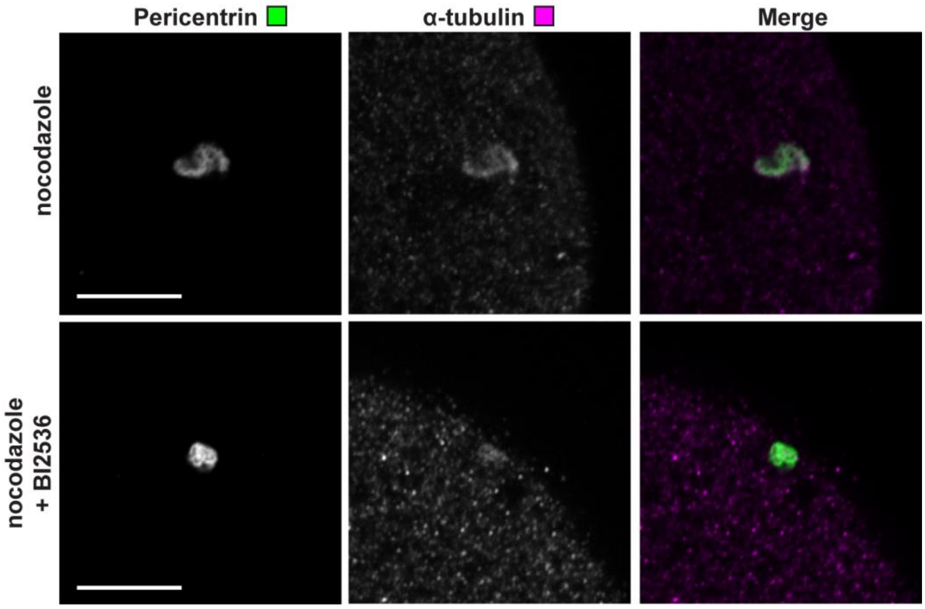

Supplementary Figure 8 Microtubules are absent following nocodazole treatment

Oocytes treated with nocodazole or with nocodazole + BI 2536 were fixed just after NEBD and stained for Pericentrin (green, MTOCs) and α-tubulin (magenta, microtubules).

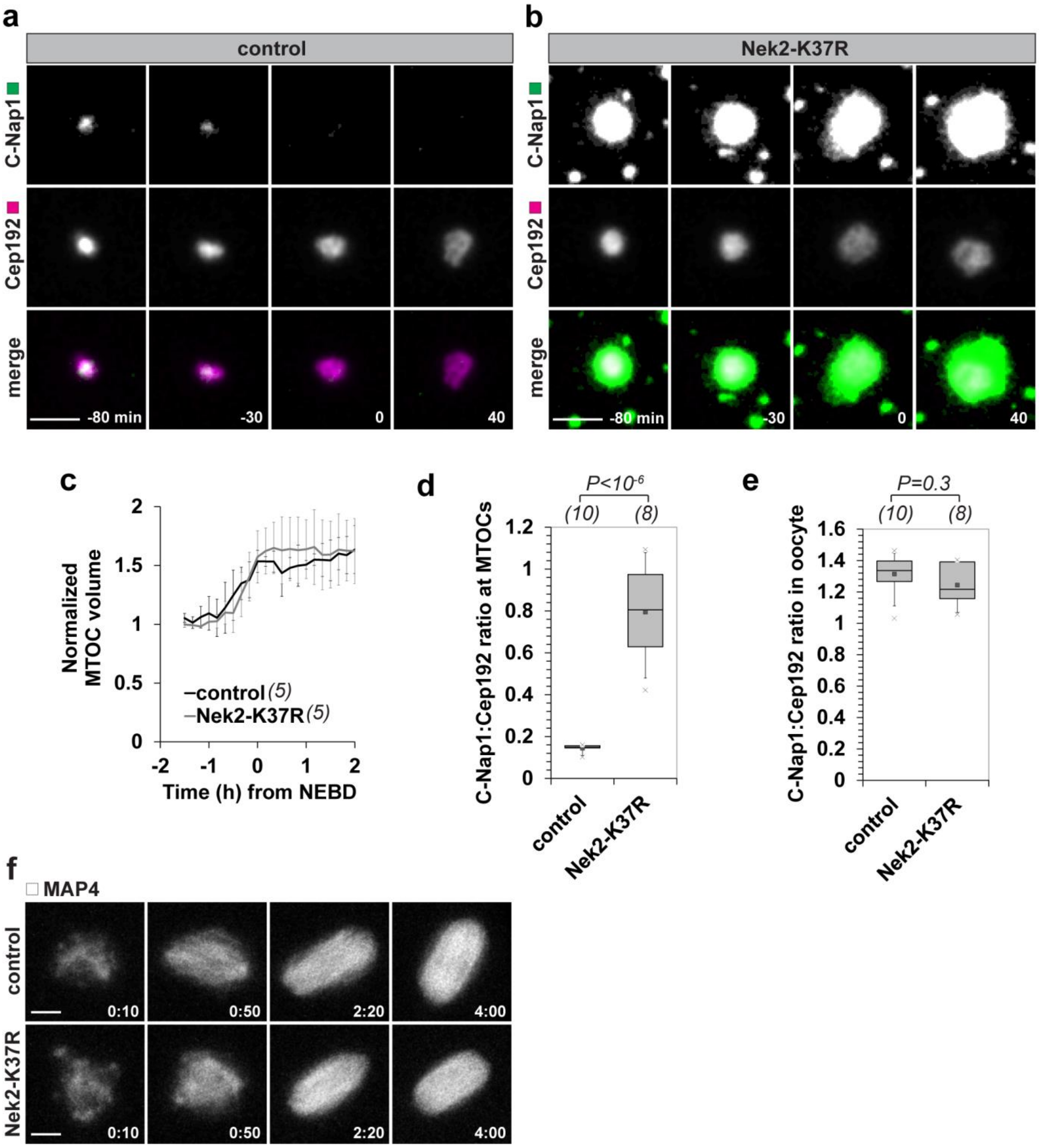

Supplementary Figure 9 Increased C-Nap1 levels at MTOCs does not perturb MTOC decondensation or spindle assembly

(a,b) 3D Time-lapse imaging of live mouse oocytes treated with nocodazole expressing mEGFP-C-Nap1 (green) and mCherry-Cep192 (magenta, MTOCs) injected with either **(a)** water (control) or **(b)** Nek2-K37R mRNA. Z-projection, 15 sections, every 1.5 µm. Scale bar, 5 µm. Time, minutes from NEBD. **(c)** Data sets shown in **a** and **b** were used to quantify

MTOC volume over time (normalized to initial value). Error bars show s.d.. Number of oocytes analysed for each condition is specified in brackets. **(d,e)** Prophase-arrested oocytes expressing mEGFP-C-Nap1 and mCherry-Cep192 were injected with either water (control) or Nek2-K37R mRNA. Box plots show **(d)** the ratio of mean C-Nap1 intensity to mean Cep192 intensity within the MTOC region and **(e)** the ratio of mean C-Nap1 intensity to mean Cep192 intensity within the entire oocyte 3 hours after Nek2-K37R mRNA microinjection. Number of oocytes analysed for each condition is specified in brackets above each box. *P* values were calculated with Student's *t*-test. **(f)** 3D Time-lapse imaging of live mouse oocytes expressing either mCherry-MAP4 (control) or mCherry-MAP4 and Nek2-K37 (Nek2-K37R). Z-projection, 9 sections, every 3 μm. Scale bar, 10 μm. Time, minutes from NEBD.

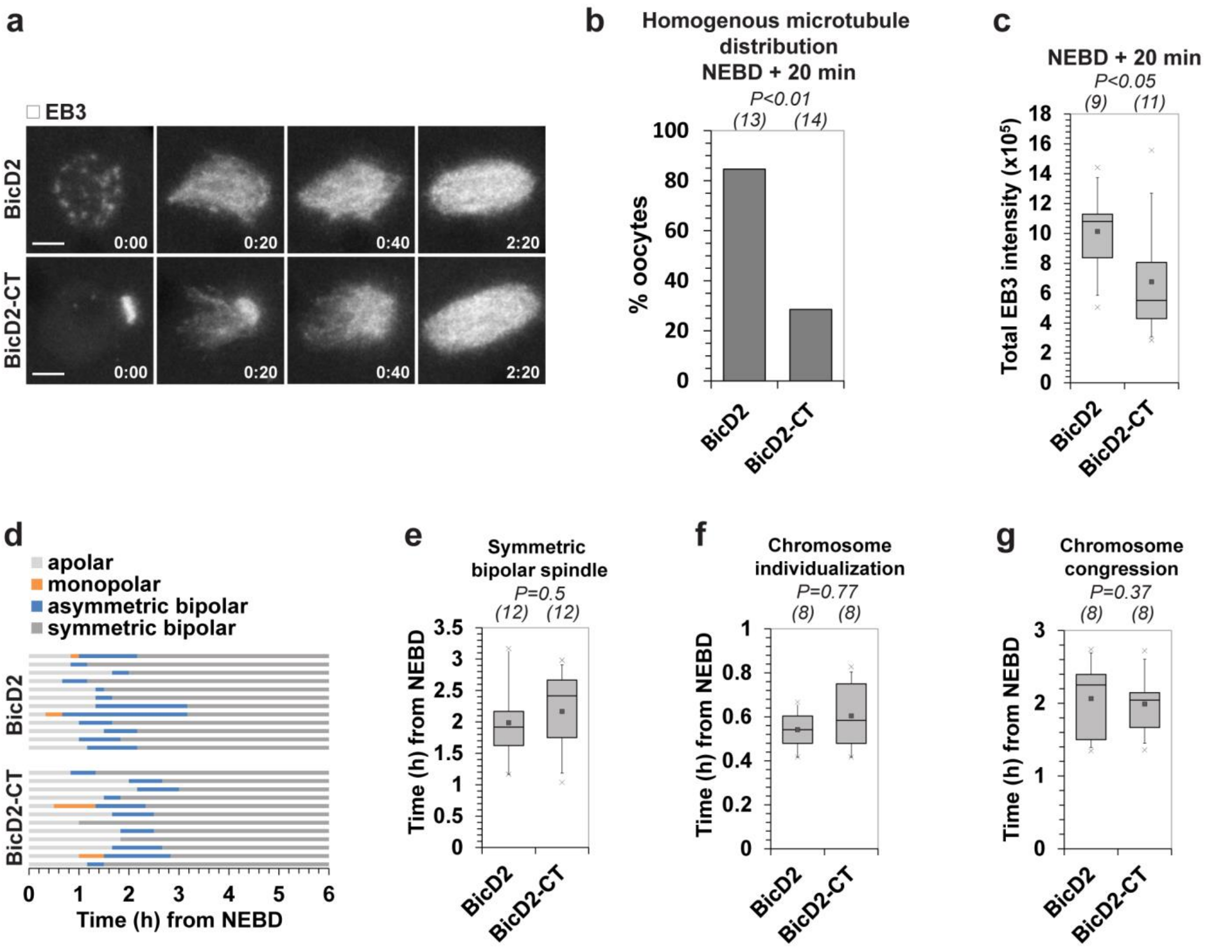

Supplementary Figure 10 MTOC stretching prior to NEBD is not required for timely spindle assembly and chromosome congression

(a) 3D Time-lapse imaging of live mouse oocytes of expressing EB3-mCherry and either BicD2 or BicD2-CT. Scale bar, 10 µm. Time, minutes from NEBD. Representative examples. More than 9 oocytes analysed for each condition. **(b)** Oocytes were scored for homogeneous distribution of microtubules (EB3-mCherry) 20 minutes after NEBD. Number of oocytes analysed for each condition is specified in brackets above each column. *P* value was calculated with Fisher's exact test. **(c)** Box plots of total EB3 intensity at 20 minutes after NEBD . *P* value was calculated with Student's *t*-test. **(d)** Stages of spindle assembly quantified from oocytes expressing KIF11-mEGFP and either mCherry-BicD2 (BicD2) or mCherry-BicD2-CT (BicD2-CT) are plotted for individual oocytes (each bar) over time.

Presence of a spindle pole was determined as a focused accumulation of KIF11-mEGFP signal. Aggregation over 2 independent experiments. **(e)** Box plots showing time of symmetric bipolar spindle formation quantified from oocytes in **a**. Number of oocytes analysed for each condition is specified in brackets (aggregation over 2 independent experiments). *P* values were calculated with Student's *t*-test. **(f,g)** Box plots showing time of **(c)** chromosome individualization and **(d)** chromosome congression quantified from oocytes expressing H2B-mRFP1 and either mEGFP-BicD2 (BicD2) or mEGFP-BicD2-CT (BicD2-CT). Number of oocytes analysed for each condition is specified in brackets. *P* values were calculated with Student's *t*-test.

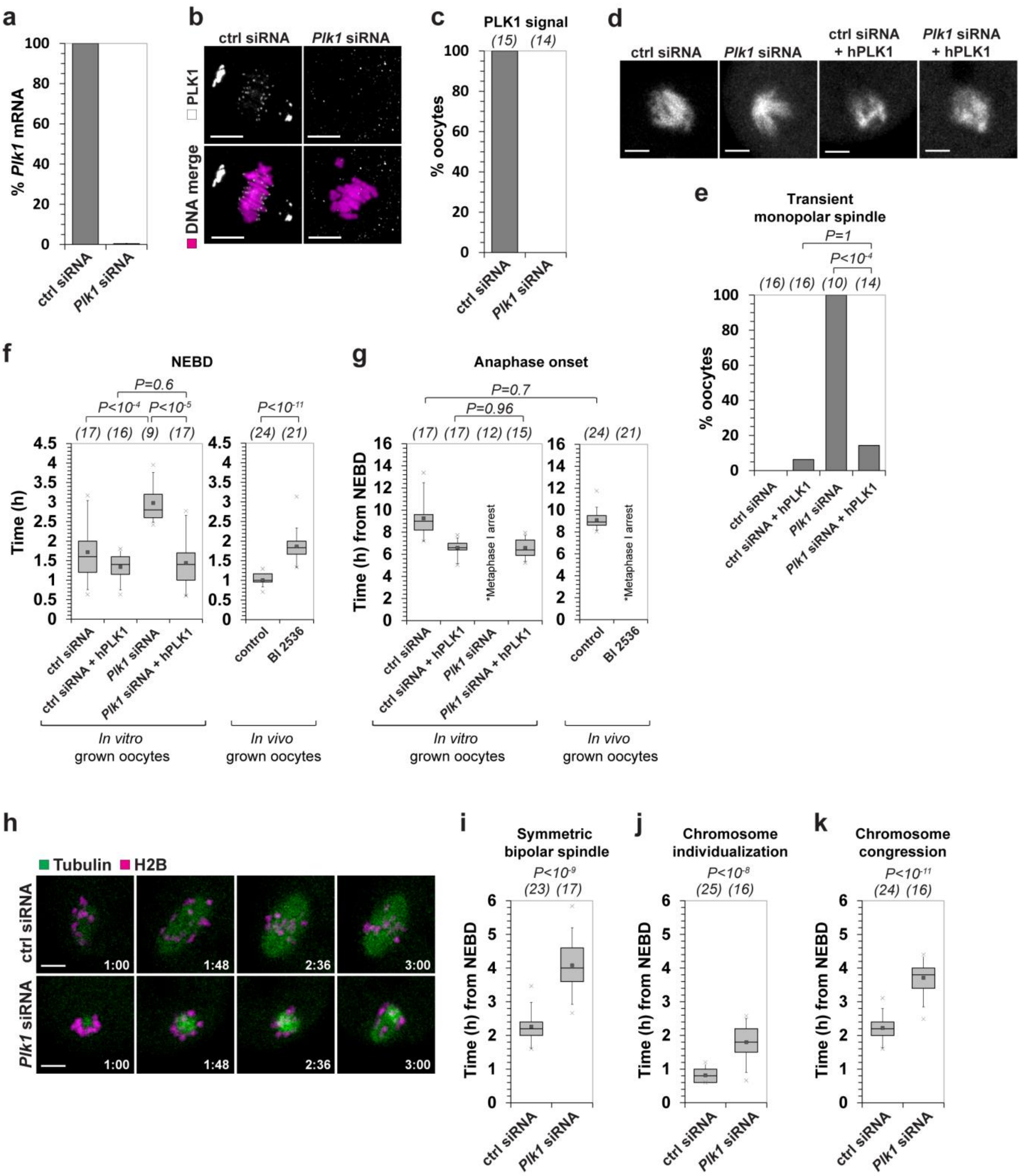

Supplementary Figure 11 PLK1 knockdown phenocopies BI 2536 treatment and can be rescued with human PLK1

(a) *Plk1* mRNA levels were determined by RT-qPCR from oocytes injected with control siRNAs (ctrl siRNA) and *Plk1* siRNAs (*Plk1* siRNA). *Plk1* mRNA levels were normalized to ctrl siRNA oocytes. **(b)** Ctrl siRNA and *Plk1* siRNA oocytes were fixed 6hr after NEBD and

stained for PLK1 (grey) and DNA (magenta). Z-projection, 28 sections, every 0.5 μm. Scale bar, 10 μm. **(c)** oocytes from **b** were scored for PLK1 signal at kinetochores and MTOCs. Number of oocytes analysed for each condition is specified in brackets. **(d)** Images of microtubules (mCherry-MAP4) 36 minutes after NEBD in live oocytes injected with control siRNA (ctrl siRNA), control siRNA + mEGFP-hPLK1 (ctrl siRNA + hPLK1), *Plk1* siRNAs (*Plk1* siRNA), and *Plk1* siRNAs + mEGFP-hPLK1 (*Plk1* siRNA + hPLK1). Representative examples shown from 2 independent experiments (>10 oocytes analysed for each condition). **(e)** Oocytes were scored for the presence of transient monopolar spindles. Number of oocytes analysed for each condition is specified in brackets above each column (aggregation over 2 independent experiments). *P* value was calculated with Fisher's exact test. **(f,g)** Box plots showing **(f)** time of NEBD following dbcAMP wash-out and **(g)** time of anaphase onset. Oocytes used for siRNA experiments were grown *in vitro* within follicles to allow PLK1 depletion (*in vitro* grown oocytes), see methods. Oocytes treated with DMSO (control) and BI 2536 were isolated directly from ovaries (*in vivo* grown oocytes), see methods. Number of oocytes analysed for each condition is specified in brackets above each box (aggregation over 2-4 independent experiments). *P* values were calculated with Student's *t*-test. **(h)** 3D Time-lapse imaging of live ctrl siRNA and *Plk1* siRNA oocytes expressing mEGFP-α-Tubulin (green, microtubules) and H2B-mRFP1 (magenta, chromosomes). Scale bar, 10 μm. Time, minutes from NEBD. Representative examples from 2 independent experiments (>15 oocytes total for each condition). **(i-k)** Box plots showing **(i)** time of symmetric bipolar spindle formation, **(j)** chromosome individualization and **(k)** chromosome congression quantified from oocytes in **h**. Number of oocytes analysed for each condition is specified in brackets (aggregation over 2 independent experiments). *P* values were calculated with Student's *t*-test.

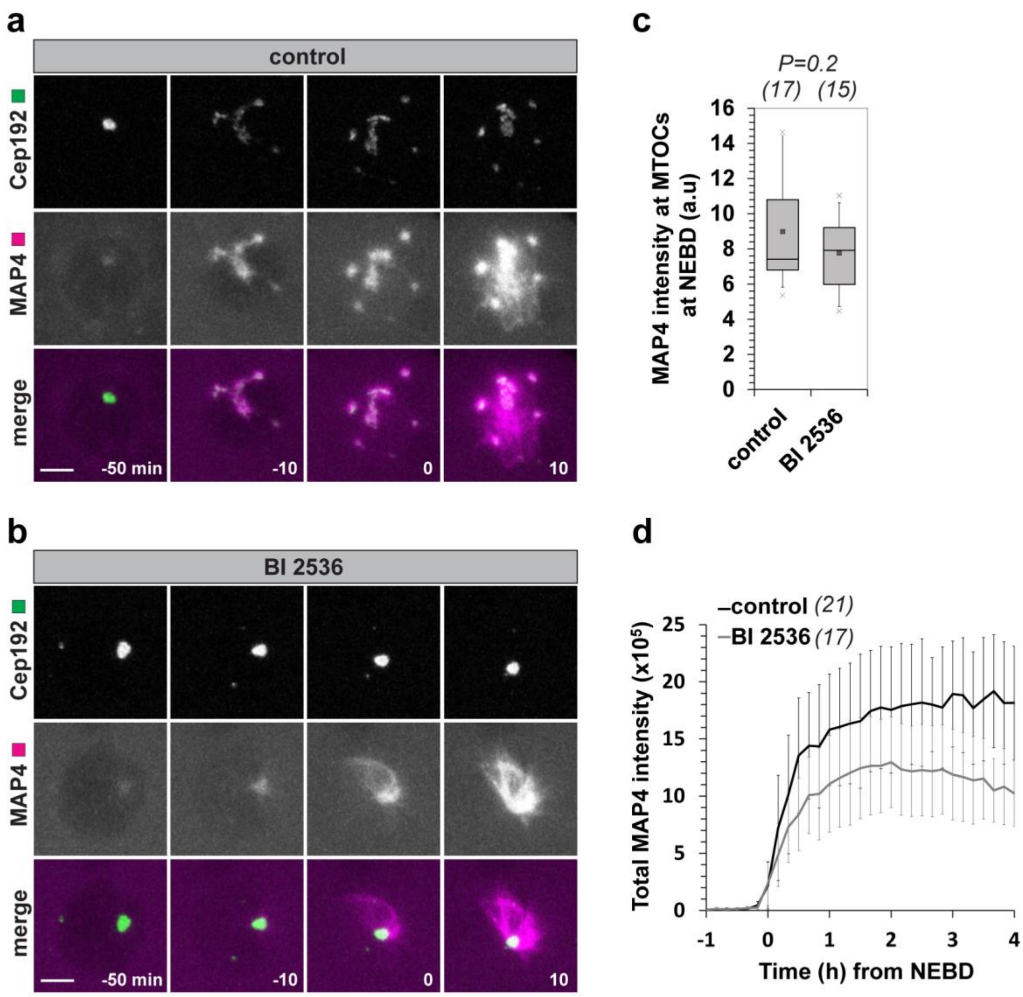

Supplementary Figure 12 Microtubule nucleation when PLK1 is inhibited

(a,b) 3D Time-lapse imaging of live mouse oocytes expressing EGFP-Cep192 (green, MTOCs) and mCherry-MAP4 (magenta, microtubules) treated with **(a)** DMSO (control) or **(b)** BI 2536. Z-projection, 25 sections, every 1.5 μm. Scale bar, 10 μm. Time, minutes from NEBD. Representative examples from 3 independent experiments (>15 oocytes total for each condition). **(c)** Box plots of the mean MAP4 intensity within the MTOC region at NEBD. Number of oocytes analysed for each condition is specified in brackets. *P* value was calculated with Student's *t*-test. **(d)** Total MAP4 intensity plotted over time. Number of oocytes analysed for each condition is specified in brackets. Error bars show s.d..
